# Unravelling HCV diversity and resistance in Viet Nam: a cross-sectional analysis

**DOI:** 10.1016/j.lanwpc.2026.101956

**Published:** 2026-08-19

**Authors:** Chau Le Ngoc, Haiting Chai, George Airey, Tanmay Das, Daisy Jennings, Fuqing Xu, Barnaby Flower, Mahdi Moradi Marjaneh, Leanne McCabe, Hung Le Manh, Chau Nguyen Van Vinh, Thuan Dang Trong, Thach Pham Ngoc, Huong Vu Thi Thu, Guy E. Thwaites, H. Rogier van Doorn, Jeremy Day, Evelyne Kestelyn, Tan Le Van, Motiur Rahman, Sarah Pett, Eleanor Barnes, A. Sarah Walker, Graham S. Cooke, M. Azim Ansari

**Affiliations:** a Oxford University Clinical Research Unit (OUCRU), Ho Chi Minh City, Viet Nam; b Department of Infectious Disease, Imperial College London, London, UK; c NIHR BRC Imperial College NHS Trust, London, UK; d MRC Clinical Trial Unit at UCL, University College London, London, UK; e Hospital for Tropical Diseases, Ho Chi Minh City, Viet Nam; f Nuffield Department of Medicine, University of Oxford, Oxford, UK; g NIHR BRC Oxford University NHS Trust, Oxford, UK; h National Hospital for Tropical Diseases, Ha Noi, Viet Nam

**Keywords:** Hepatitis C, Genotype, Subtype, Resistance-associated substitution, Direct-acting antiviral, Viet Nam

## Abstract

**Background:**

Viet Nam has one of the world's most diverse hepatitis C virus (HCV) epidemics, dominated by genotype 6. Understanding pre-treatment resistance-associated substitutions (RASs) particularly in under-studied genotype 6 is important for informing resistance surveillance and supporting national HCV elimination strategies. We aimed to evaluate the landscape of viral diversity and baseline RASs relevant to currently used direct-acting antivirals (DAAs) in Viet Nam.

**Methods:**

We utilised whole-genome sequencing to analyse HCV isolates from a cohort of 1649 treatment-naïve patients enrolled in six clinical studies in Viet Nam between 2013 and 2023. The study assessed genotype and subtype distribution, associations with demographic and clinical variables, and prevalence of known and putative RASs in NS3, NS5A, and NS5B relevant to DAAs used in Viet Nam.

**Findings:**

Phylogenetic analysis revealed that genotype 6 was dominant (50.3%, 829/1649). We observed distinct geographical and demographic partitioning: genotype 2 was concentrated in the south and associated with older age and HIV co-infection, while genotype 3 was clustered in the north among younger males. Clinically relevant RASs were detected in 37.9% (617/1630) of patients, with the highest burden in NS5A region. Genotypes 2 and subtype 3b demonstrated a high prevalence of subtype-specific polymorphisms occurring at previously reported resistance-associated positions. Among genotype 6 infections, subtype 6a frequently carried L28F mutation (43.3%, 181/418), whereas subtype 6e demonstrated a low prevalence of previously reported clinical RASs.

**Interpretation:**

Viet Nam is characterised by a complex, genotype 6-predominant HCV epidemic with substantial subtype-specific baseline resistance diversity. The high prevalence of subtype-defining polymorphisms observed in several genotype 2 and subtype 3b lineages highlights the importance of continued molecular surveillance and may have implications for optimisation of DAA regimen selection and resistance monitoring strategies within highly diverse HCV populations.

**Funding:**

Wellcome Trust.

**Research in context**

**Evidence before this study**

Viet Nam bears one of the world's most genetically complex hepatitis C virus epidemics, with genotype 6 (under-represented in global clinical trials) accounting for >50% of infections. We systematically reviewed PubMed, Embase, and Web of Science from January 2000 to July 2026 using terms including “hepatitis C”, “genotype 6”, “Viet Nam”, “resistance-associated substitutions” and “direct-acting antivirals”. Existing studies revealed critical gaps: most were limited to small cohorts, partial genomic regions (typically NS5A or NS5B only), or single subtypes. Crucially, no large-scale whole-genome study had comprehensively characterised baseline resistance-associated substitutions across the diverse HCV genotype 6 subtypes circulating in Viet Nam, a major concern as the Western Pacific region pursues WHO 2030 elimination targets amid expanding DAA access. We also searched NCBI GenBank for HCV whole genomes and found only 57 whole genomes available from Viet Nam with 55 of those being genotype 6.

**Added value of this study**

We present one of the largest whole-genome sequencing studies of HCV reported from Southeast Asia (1649 isolates, 2013–2023), significantly increasing the available genotype 6 genomes globally. Our analysis reveals three findings relevant for regional elimination efforts: (1) High prevalence of subtype-defining polymorphisms at previously reported resistance-associated positions: Near-fixation of NS5A polymorphisms in genotypes 2 (L31M in 98.1% of subtype 2m) and 3b (A30K + L31M dual mutation in 90.0%) corresponded to >90% predicted resistance prevalence for several NS5A inhibitors. These findings highlight substantial subtype-specific differences in predicted NS5A-associated resistance profiles. (2) Critical subtype heterogeneity: Within the dominant genotype 6, we observed marked differences in baseline RAS profiles between subtypes, with subtype 6a frequently harbouring the L28F substitution (43.3%), corresponding to a higher predicted prevalence of daclatasvir-associated RASs than subtype 6e; whereas subtype 6e showed minimal resistance-associated substitutions, highlighting the genetic diversity that exists even within a single genotype; (3) Geographic-demographic partitioning: Distinct transmission networks are evident, with genotype 2 concentrated among older women in the Mekong Delta and genotype 3b clustered among young men in the north, providing epidemiological information that may help inform targeted screening and surveillance strategies.

**Implications of all the available evidence**

These findings demonstrate the substantial genetic diversity of HCV circulating in Viet Nam together with marked subtype-specific variability in baseline RAS prevalence, supporting continued molecular surveillance in regions where genetically diverse viral populations predominate. For Viet Nam, where DAAs have been national policy since 2016 but elimination progress lags, our data argue for: (1) Enhanced genomic surveillance to inform treatment guidelines, with particular emphasis on the need for further phenotypic studies and prospective clinical outcome studies to determine the clinical significance of the subtype-specific polymorphism patterns identified in this study, particularly among genotype 2 subtypes and subtype 3b; (2) Strategic deployment of resources based on geographic resistance patterns, prioritising enhanced surveillance in regions with high concentrations of subtypes (Mekong Delta for genotype 2, northern provinces for genotype 3b) to guide evidence generation.

## Introduction

Hepatitis C virus infection represents a significant global health challenge, being a primary cause of chronic liver disease, which often progresses to severe outcomes like cirrhosis and hepatocellular carcinoma over decades. The World Health Organisation estimated that in 2022, approximately 50 million people lived with chronic HCV globally, with about 1 million new infections each year.^1^ The burden varies significantly by region. In Southeast Asia, and specifically Viet Nam, prevalence estimates range from 1% to 4.7% in the general population but can be as high as 89% within high-risk groups, including people who inject drugs (PWIDs), people living with HIV (PLWH), and those frequently receiving blood transfusions or dialysis.^2^, ^3^, ^4^

HCV is an enveloped, positive-sense single-stranded RNA virus of the Flaviviridae family. Its genome (∼9.6 kb) encodes a single polyprotein processed into three structural (core, E1, E2) and seven non-structural proteins (p7, NS2, NS3, NS4A, NS4B, NS5A, NS5B). It has been classified into eight genotypes^1^, ^2^, ^3^, ^4^, ^5^, ^6^, ^7^, ^8^ and over 100 subtypes, with genotype and subtype influencing disease progression and response to treatment.^5^ Globally, genotype 1 (46.2%) and genotype 3 (30.1%) are most common.^6^ However, Viet Nam and surrounding Southeast Asian nations exhibit a distinct epidemiological landscape dominated by genotype 6, which accounts for over 50% of infections, particularly in southern and central areas, followed by genotypes 1 and 2.^7^^,^^8^

Transmission primarily occurs via blood-to-blood contact. Injection drug use is a major global driver, alongside historical transmission through unsafe blood transfusions prior to widespread screening. Across resource-limited settings, traditional practices like tattooing or acupuncture with non-sterile tools have also been linked to transmission.^4^^,^^9^^,^^10^

Genotype 6, notable for its high genetic diversity with over 30 subtypes, likely originated in Southeast Asia and remains largely concentrated there. In Viet Nam, genotype 6 constitutes 54.4% of cases, mainly in the southern and central regions, with subtypes 6a, 6e, 6h, and 6l being prominent.^11^ Phylogenetic studies suggest Viet Nam as a potential source for genotype 6 isolates spreading regionally and globally via migration.^2^^,^^7^^,^^8^ Transmission patterns within Viet Nam also show genotype specificity: genotype 1 is strongly linked to PWIDs (up to 70.1% prevalence in this group), whereas genotype 6 is more frequently detected among dialysis patients and those with multiple transfusions, suggesting nosocomial spread.^12^ These complex dynamics, influenced by healthcare practices, risk behaviours, and population movement, echo trends seen in neighbouring regions.^11^^,^^13^

The landscape of HCV treatment has transformed since the discovery of virus in 1989. Early interferon-based therapies offered limited sustained virologic response (SVR) rates and significant side effects. The advent of direct-acting antivirals, starting with first-generation agents like boceprevir and telaprevir in 2011 marked a turning point. Second-generation DAAs, such as ledipasvir and sofosbuvir (available by 2013–2014), have enabled highly effective (>90% SVR), shorter, safer, and interferon-free regimens. Viet Nam officially adopted DAAs as first-line treatment in 2016, leading to dramatically improved cure rates, although equitable access remains a challenge.^14^

Despite the success of DAAs, the pre-existence or emergence of resistance-associated substitutions poses a significant threat to treatment efficacy. RASs are mutations that decrease drug susceptibility, often quantified *in vitro* by an increased half-maximal effective concentration (EC50). Key examples include substitutions at positions D168 (NS3), Y93 (NS5A), S282, and C316 (NS5B). These substitutions can exist as natural variants within the viral population or emerge under the selective pressure of drug treatment, potentially resulting in treatment failure, particularly in patients with prior DAA exposure, poor adherence, or advanced liver disease.^15^, ^16^, ^17^ The prevalence and clinical impact of RASs vary considerably by HCV genotype. While global analyses suggest genotype 6 has the highest RAS prevalence, detailed data, particularly at the subtype level, are scarce.^18^ This knowledge gap hinders the development of optimised treatment strategies for genotype 6, which is critically important in Viet Nam given its prevalence.^11^^,^^18^

Therefore, continuous RAS surveillance is essential, particularly in regions like Southeast Asia with high genotype diversity. Existing studies on baseline RASs in treatment-naïve Vietnamese patients are limited and often focus only on a specific region like NS5B.^19^^,^^20^ Comprehensive data on the prevalence and patterns of RASs across different genotypes, subtypes, and DAA target regions in Viet Nam are urgently needed. Understanding these patterns is critical for informing clinical practice, guiding retreatment strategies, shaping national HCV management policies, and ultimately achieving HCV elimination goals. This study aimed to understand the prevalence of different HCV genotypes in Viet Nam and their geographical and demographic associations and to characterise the prevalence of pre-existing RASs across a large cohort of diverse HCV isolates collected throughout Viet Nam.

## Methods

### Study description

Plasma samples were obtained from a total of 1651 patients confirmed to be positive for Hepatitis C virus. These patients were enrolled in six distinct clinical research studies (VHARP001, VIZIONS, 01EI, KAPB, SEARCH-1 and VIETNARMS) conducted across northern, central, and southern Viet Nam between 2013 and 2023. The initial trials from 2013 to 2020 were carried out in Ho Chi Minh city. Enrolment was expanded to include additional sites in Hanoi starting in 2020. Socio-demographic data and results from serological tests were extracted from the respective study databases. Detailed information regarding study periods, inclusion and exclusion criteria, enrolment sites, and HCV positive participant numbers for each clinical study is summarised in Supplementary Table S6. All plasma samples included in this study were collected at baseline prior to direct-acting antiviral exposure, and all participants were treatment-naïve at the time of sample collection.

### HCV RNA extraction

Viral RNA was extracted from 200 μl of plasma using the QIAamp Viral RNA Mini Kit (Qiagen, Germany), with linear acrylamide added as a carrier. RNA was eluted in 30 μl of buffer AVE and stored at −80 °C until further processing.

### Library preparation and next-generation sequencing (NGS)

Double-stranded cDNA was generated using the NEBNext® Ultra™ II Directional RNA Library Prep Kit for Illumina (New England Biolabs, USA), following manufacturer guidelines with random priming and strand-specific synthesis. Purified cDNA was obtained using SPRIselect magnetic beads (Beckman Coulter, USA). Sequencing libraries were prepared from purified double-stranded cDNA using the NEBNext® Ultra™ II Directional RNA Library Prep Kit and NEBNext® Multiplex Oligos for Illumina® (New England Biolabs, USA), including end repair, adaptor ligation, and PCR enrichment. Final libraries were purified using SPRIselect beads and assessed for fragment size and concentration using the Agilent TapeStation (Agilent Technologies, USA) and Qubit fluorometry (Thermo Fisher Scientific, USA). Target enrichment was performed using the Twist Hybridisation and Wash Kit with Amplification Mix (Twist Bioscience, USA) and a custom probe panel, followed by post-capture PCR amplification. Final libraries were quantified, normalised, and sequenced on the Illumina MiSeq platform according to the manufacturer's instructions.

### Sanger sequencing of samples without NGS genome recovery

Samples that did not yield useable genome sequences by NGS due to insufficient coverage were subjected to targeted Sanger sequencing. Viral RNA was reverse transcribed to cDNA using SuperScript III reverse transcriptase protocol as described earlier,^2^ and specific genomic fragments were amplified using sets of primers designed across the HCV genome (full list in Supplementary Table S7). PCR products were purified and sequenced using the Sanger method on an ABI 3130xl DNA Analyser (Applied Biosystems, USA) according to the manufacturer's instructions. Resulting chromatograms were inspected for sequence quality, and consensus sequences were generated and aligned to the H77 reference genome (GenBank accession: AY051292.1) to determine genomic positions and confirm subtype assignments.

### Identification of previously reported and novel resistance-associated substitutions

Raw sequencing reads generated on the Illumina MiSeq platform were processed using QuasR (v1.01)^21^ to remove low-quality reads (length <50 bp or median Phred quality < Q30) and cutadapter (v1.7.1)^22^ to trim Illumina sequencing adaptors.^23^ Human-derived reads were removed by aligning against the T2T human genome assembly via bowtie2 (v2.2.4).^24^^,^^25^ Viral genomes were then assembled using bwa (v0.7.17)^26^ and VICUNA (v1.3)^27^ based on the refined reads and HCV references from International Committee on Taxonomy of Viruses (ICTV).^28^ Consensus nucleotides were called with SHIVER (v1.0.7),^29^ requiring support from at least 50% of aligned reads at each position. Genome coordinates are reported relative to the H77 reference sequence. For each genomic position analysed, only sequences with sufficient sequencing coverage and a valid amino acid call at that site were included in the denominator; sequences failing coverage thresholds at the corresponding position were excluded from site-specific analyses.

Following an extensive literature review, previously reported resistance sites associated with DAA treatment outcomes were divided into three categories for the purpose of this study. Our analysis focused on substitutions relevant to the DAAs approved or used in Viet Nam from 2016 to 2024, specifically glecaprevir, grazoprevir, voxilaprevir, daclatasvir, elbasvir, ledipasvir, pibrentasvir, velpatasvir, and sofosbuvir.

#### Clinical resistance-associated substitutions (Clinical RASs)

These are amino acid variants at well-characterised NS3, NS5A, and NS5B positions that have been demonstrated *in vitro* or in clinical studies to confer a measurable and clinically meaningful reduction in DAA susceptibility. Their presence is associated with virologic failure, reduced treatment efficacy, or necessitates a regimen adjustment in clinical guidelines (full list in Supplementary Figure S1).

#### Sub-clinical resistance-associated substitutions (Sub-clinical substitutions)

These refer to previously reported RASs that are generally not considered clinically significant or are believed to have a minimal impact on drug resistance. These were classified within the context of DAAs used in Viet Nam.

#### Putative new RASs

We systematically screened for novel amino acid variants detected at the same genomic positions previously characterised as clinically relevant RAS sites. Specifically, any substitution known to confer resistance in other HCV subtypes but not yet documented in the subtype under investigation was classified as a putative new RAS candidate. These variants represent mutations that may potentially influence DAA susceptibility and were flagged as targets for future phenotypic evaluation to verify their functional impact.

### Data analysis

All statistical analyses were performed using R in RStudio (R Version 4.3.3; RStudio, PBC, Boston, MA, USA).

#### Geospatial analysis

To visualise the geographical distribution of HCV genotypes across Viet Nam, geospatial analyses were performed using aggregated geographic information at the city or province level. Latitude and longitude coordinates assigned to each sample corresponded to the participant's reported city or province of enrolment and were used solely for regional-level mapping and visualisation. These data were mapped using administrative boundary shapefiles from the Global Administrative Areas database (GADM) version 4.1. All geospatial analyses were conducted using de-identified study IDs to preserve participant anonymity and confidentiality.

#### Statistics

The study cohort was initially characterised using descriptive statistics (median and interquartile range for continuous variables, and counts and percentages for categorical variables), summarising baseline characteristics such as sex, age at enrolment, HCV RNA viral load, cirrhosis status, and HIV/HBV co-infection, stratified by HCV genotype. For inferential statistics, due to non-normality of distributions, age and HCV RNA viral load were compared between genotypes using non-parametric Kruskal–Wallis test, and categorical variables were compared using Chi-squared test.

#### Statistical significance

For all statistical tests, a p-value less than 0.05 was considered statistically significant.

### Ethics

This cross-sectional analysis used clinical samples and associated metadata collected prospectively through six previously approved HCV clinical studies conducted by the Oxford University Clinical Research Unit (OUCRU) in collaboration with the Hospital for Tropical Diseases, Ho Chi Minh City, Viet Nam, and collaborating institutions. The VHARP001 study was approved by the Oxford Tropical Research Ethics Committee (OxTREC; 36-10), the Ministry of Health of Viet Nam (3277/QD-BYT), and the Ethics Committee of the Hospital for Tropical Diseases, Ho Chi Minh City (24/BVBND-KHTH). The VIZIONS study was approved by the Oxford Tropical Research Ethics Committee (OxTREC; 15-12), the Ethics Committee of the Hospital for Tropical Diseases, Ho Chi Minh City (136/BVBND-KH), the Ethics Committee of the National Hospital for Tropical Diseases (38/HDDD-NDTU), Dong Thap General Hospital (821 A/QD-BVDT-TCCB), Khanh Hoa General Hospital (365/BVDKT), Dak Lak General Hospital (489/BVT-KHTH), and Hue Central Hospital (77/BVTUH). The 01EI study was approved by the Oxford Tropical Research Ethics Committee (OxTREC; 152-12) and the Ethics Committee of the Hospital for Tropical Diseases, Ho Chi Minh City (442/BVBND). The KAPB study was approved by the Oxford Tropical Research Ethics Committee (OxTREC; 44-17) and the Ethics Committee of the Hospital for Tropical Diseases, Ho Chi Minh City (22/HDDD). The SEARCH-1 study was approved by the Oxford Tropical Research Ethics Committee (OxTREC; 43-17), the Imperial College Research Ethics Committee (17IC4238), the Ministry of Health of Viet Nam (78/CN-HDDD), and the Ethics Committee of the Hospital for Tropical Diseases, Ho Chi Minh City (31/HDDD). The VIETNARMS study was approved by the Oxford Tropical Research Ethics Committee (OxTREC; 9-19), the Imperial College Research Ethics Committee (18IC4919), the Ministry of Health of Viet Nam (89/CN-HDDD), the Ethics Committee of the National Hospital for Tropical Diseases (34/HDDD-NDTU), and the Ethics Committee of the Hospital for Tropical Diseases, Ho Chi Minh City (29/HDDD). Written informed consent was obtained from all participants before enrolment in the respective studies.

### Role of the funding source

This study was funded by Wellcome Trust (award number 206296/Z/17/Z). The funding source had no role in the study design, data collection, data analysis, data interpretation, writing of the manuscript, or the decision to submit the manuscript for publication.

## Results

### Prevalence and diversity of HCV genotypes and subtypes in Viet Nam

Successful whole-genome sequencing was achieved for 1649 HCV isolates derived from 1651 collected samples. Among these, 1285 sequences were generated using next-generation sequencing and 364 were obtained by Sanger sequencing. Phylogenetic analysis indicated that genotype 6 was the most prevalent, identified in 829 sequences (50.3%). This genotype exhibited significant diversity, with subtype 6a being the most prevalent (28.4%, 468/1649), followed by subtype 6e (14.7%, 243/1649). Notably, subtypes 6a and 6e combined accounted for more than 40% of all sequenced samples. Other subtypes, including 6h, 6l, and 6o, were detected less frequently, while sporadic cases (fewer than 10 samples each) of 6c, 6i, 6k, 6p, 6q, 6t, 6u, and 6xb were also observed. Comparison with global reference sequences confirmed the placement of Vietnamese isolates within established HCV genotype and subtype lineages and highlighted the extensive subtype diversity present within genotype 6, providing broader phylogenetic context for the diversity observed within this cohort (Supplementary Figure S2).

Genotype 1 was the second most common genotype, comprising 649 sequences (39.4%). Within this genotype, subtypes 1a (20.5%, 338/1649) and 1b (18.9%, 311/1649) presented in nearly equal proportions. Genotype 2 accounted for 140 sequences (8.5%), primarily represented by subtypes 2a (4.3%, 71/1649) and 2m (3.6%, 59/1649). Genotype 3 was the least common, detected in only 31 sequences (1.9%), and consisted of subtypes 3b (1.3%, 21/1649) and 3a (0.6%, 10/1649). In summary, four of the eight HCV genotypes were detected in this cohort, with genotypes 6 and 1 predominating (Supplementary Figure S3).

### Regional distribution of HCV genotypes and subtypes

Patient enrolment was predominantly conducted in southern Viet Nam (n = 1270), with smaller cohorts contributed by the northern (n = 273) and central (n = 106) regions (Supplementary Figure S4). Genotypes 6 and 1, which cumulatively represented 89.7% of the study population, were detected commonly across all three regions (Supplementary Figure S5). Conversely, genotype 2 exhibited a distinct geographical restriction, being identified almost exclusively in southern Viet Nam. In the Mekong River Delta specifically, genotype 2 accounted for approximately 16.3% (80/491) of infections (Supplementary Table S1). Subtype analysis revealed further regional partitioning within genotype 2: subtype 2m predominated in the Mekong River Delta provinces, whereas subtype 2a was prevalent in both the South East and Mekong River Delta regions. Genotype 3 also displayed a marked geographical pattern, with significantly higher prevalence observed in northern Viet Nam compared to the central and southern regions. This northern concentration was primarily driven by subtype 3b, which constituted the majority of genotype 3 infections in the area (Supplementary Table S2).

### Demographic and clinical characteristics of HCV genotypes in Viet Nam

**Fig. 1:**
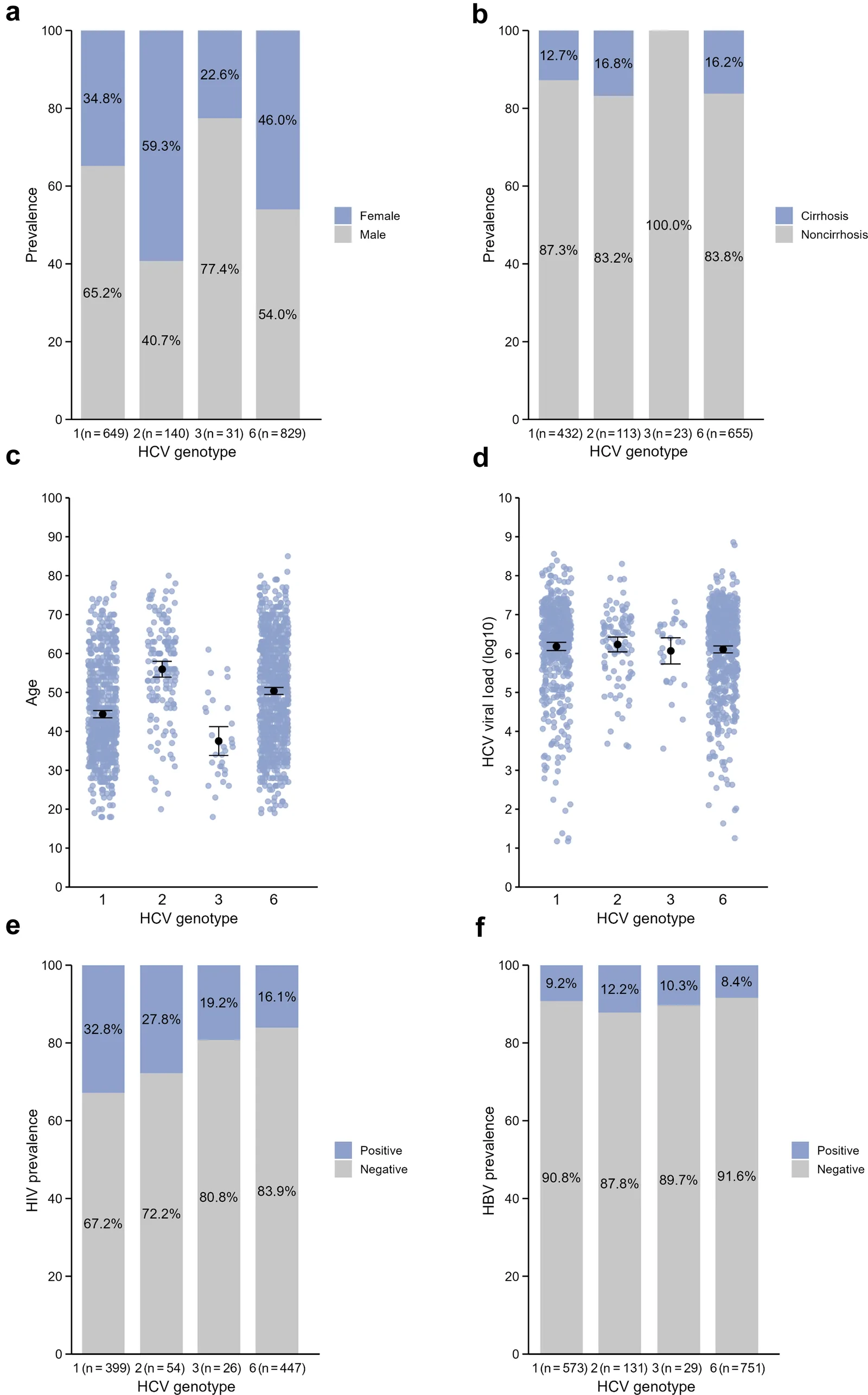
**Baseline demographic and clinical characteristics stratified by HCV genotype.** (a) Sex distribution across HCV genotypes. (b) Prevalence of cirrhosis by genotype. (c) Age at enrolment by genotype; points represent individual participants. The black dots and lines indicate mean and its 95% confidence interval. (d) Baseline HCV RNA viral load (log_10_ IU/mL) by genotype; points represent individual participants. The black dots and lines indicate mean and its 95% confidence interval. (e–f) HIV co-infection prevalence by HCV genotype (e). HBV co-infection prevalence by HCV genotype (f). Panels are restricted to individuals with available HIV status (n = 926) or HBV status (n = 1484). Abbreviations: HBV, hepatitis B virus; HCV, hepatitis C virus; HIV, human immunodeficiency virus; IU, international units.

In this Vietnamese cohort, HCV genotypes showed significant demographic differences. Sex distribution varied considerably between genotypes (p < 0.001) with genotype 3 exhibiting strong male predominance (77.4%, 24/31), while genotype 2 had a female majority (59.3%, 83/140). Genotype 1 displayed moderate male predominance (65.2%, 423/649), and genotype 6 presented a nearly balanced profile (54.0% male, 448/829). Age at enrolment also differed significantly across genotypes (p < 0.001). Patients with genotype 2 infection were on average older (median age 56 years [IQR 49–63]), those with genotype 3 infection were younger (median age 36 years [IQR 30–46]), while patients with genotypes 1 and 6 infection showed intermediate median ages (42 years [IQR 36–53] and 51 years [IQR 40–61], respectively). These patterns could potentially reflect different transmission routes or historical exposure periods (Fig. 1).

For a subset of patients, we also collected HIV and HBV co-infection status (926 patients with HIV co-infection status and 1484 patients with HBV co-infection status). HIV co-infection status showed significant association with HCV genotypes (p < 0.001), being highest among genotype 1 infections (32.8%, 131/399) and lowest among genotype 6 infections (16.1%, 72/447). In contrast, HBV co-infection showed no significant association with HCV genotypes (p = 0.51), HBV-positive prevalence was similar across HCV genotypes, observed in 12.2% (16/131) in genotype 2, 10.3% (3/29) in genotype 3, 9.2% in genotype 1 (53/573) and 8.4% in genotype 6 (63/751). Furthermore, baseline HCV RNA viral loads had a similar distribution across genotypes (6.34–6.46 log_10_ IU/ml, p = 0.36). Cirrhosis prevalence varied by genotypes (p = 0.06), being highest in genotype 2 infected patients (16.8%, 19/113), followed by genotype 6 (16.2%, 106/655) and genotype 1 (12.7%, 55/432), while no cirrhosis cases were found among the small number of genotype 3 patients (the youngest group, n = 23) (Supplementary Table S3).

### Prevalence and distribution of resistance

**Fig. 2:**
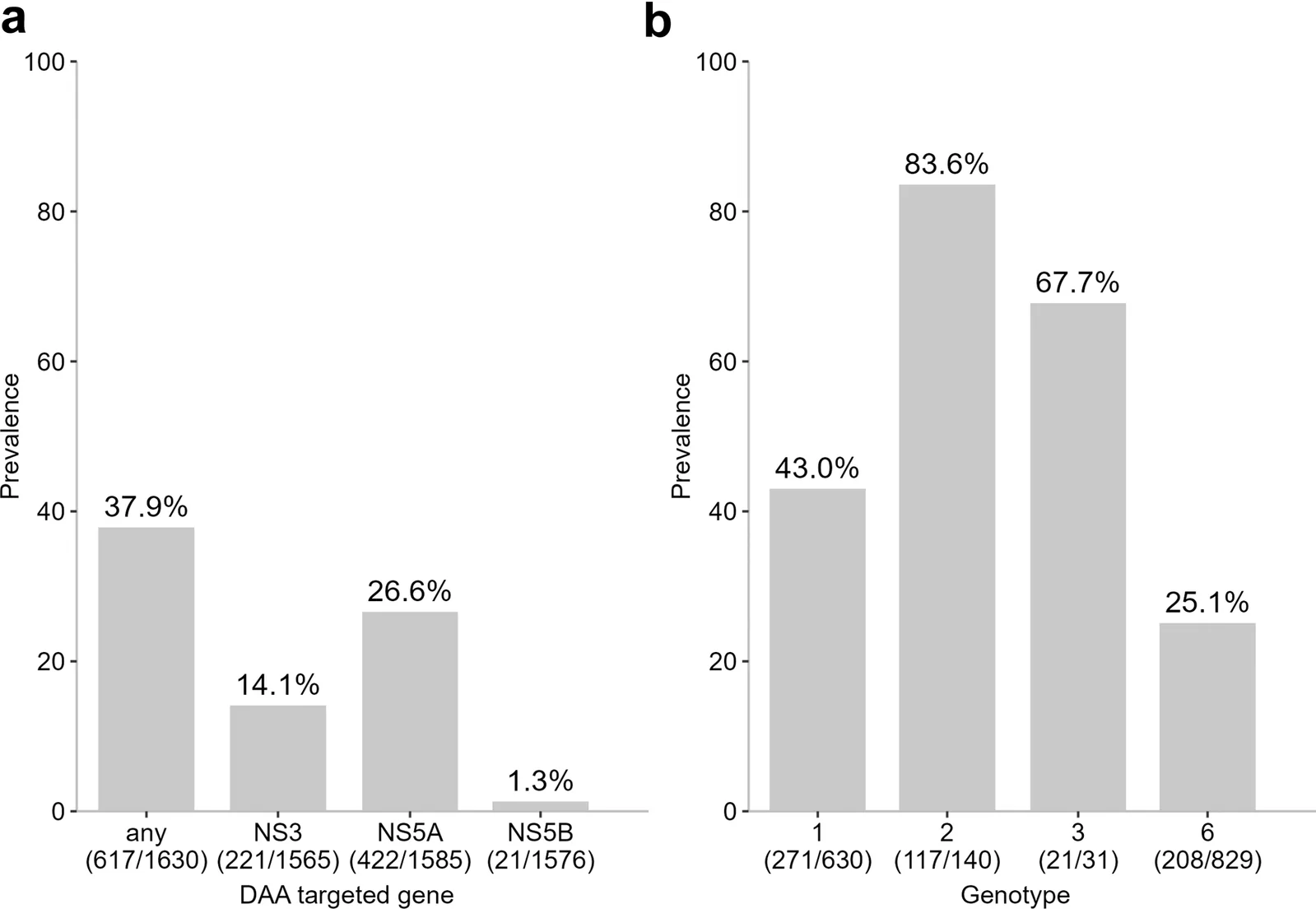
**Prevalence of pre-existing clinical RASs in the Vietnamese cohort.** (a) Distribution of RASs across the three DAA-targeted viral regions (NS3, NS5A, and NS5B). The “any” category represents the proportion of sequences harbouring at least one clinical RAS in any of the three genes. (b) Overall prevalence of RASs stratified by HCV genotype (1,2,3, and 6). The percentages above the bars indicate the prevalence of sequences containing at least one RAS, with the corresponding raw counts (number of sequences with RAS/total sequences analysed) provided below the x-axis labels.

Across this Vietnamese cohort, 37.9% (617/1630) of sequences contained at least one clinical RAS, with the NS5A protein showing the highest prevalence (26.6%, 422/1585), followed by NS3 (14.1%, 221/1565) and with very low prevalence observed for NS5B (1.3%, 21/1576). This distribution highlighted NS5A as the primary gene for clinically relevant resistance mutations (Fig. 2a). Among genotypes, clinical RAS prevalence varied significantly, with genotype 2 sequences exhibiting the highest (83.6%, 117/140), followed by genotype 3 (67.7%, 21/31), genotype 1 (43.0%, 271/630), and genotype 6 (25.1%, 208/829) (Chi-squared p < 0.001). These patterns demonstrate a markedly higher prevalence of previously reported clinical RASs among genotype 2 infections. In contrast, genotype 6, the most widely distributed genotype in Viet Nam, demonstrated the lowest overall prevalence of pre-existing clinical RASs (Fig. 2b).

#### Genotype 6, subtypes 6a and 6e

While data regarding RASs in HCV genotype 6 have historically been limited, our analysis revealed distinct resistance profiles between subtypes 6a and 6e. In subtype 6a, the RAS profile was dominated by substitutions in the NS5A region. The substitution NS5A_L28F was identified as the most prominent clinical RAS, appearing in 43.3% (181/418) of sequences. Other variants at position 28, including L28V (2.4%, 10/418), L28M (0.5%, 2/418), and L28S (0.2%, 1/418), were also detected. Additional known NS5A mutations were observed at lower frequencies, such as L31M (1.4%, 6/417) and T93S (1.9%, 8/428). This pattern of NS5A substitutions was reflected in a high predicted resistance prevalence to daclatasvir (44.5%, 186/418) and a notably lower resistance profile for velpatasvir (6.2%, 26/418), with minimal predicted resistance to elbasvir (1.4%, 6/418).

Conversely, clinical RASs in the NS3 region of subtype 6a were rare. Substitutions NS3_D168E (0.7%, 3/414) and D168Y (0.2%, 1/414) were detected at minimal frequencies, resulting in very low predicted resistance to NS3 inhibitors such as glecaprevir (0.2%, 1/421) and grazoprevir (0.9%, 4/430). Beyond clinically relevant sites, several sub-clinical RASs were observed in subtype 6a. In the NS3 region, Q41K (0.5%, 2/421) and Q41R (0.2%, 1/421) were noted, while in the NS5A region, sub-clinical variants were identified at position 24 (Q24H), 32 (P32L), 58 (T58 A/H/G/S) and position 92 (A92T).

**Fig. 3:**
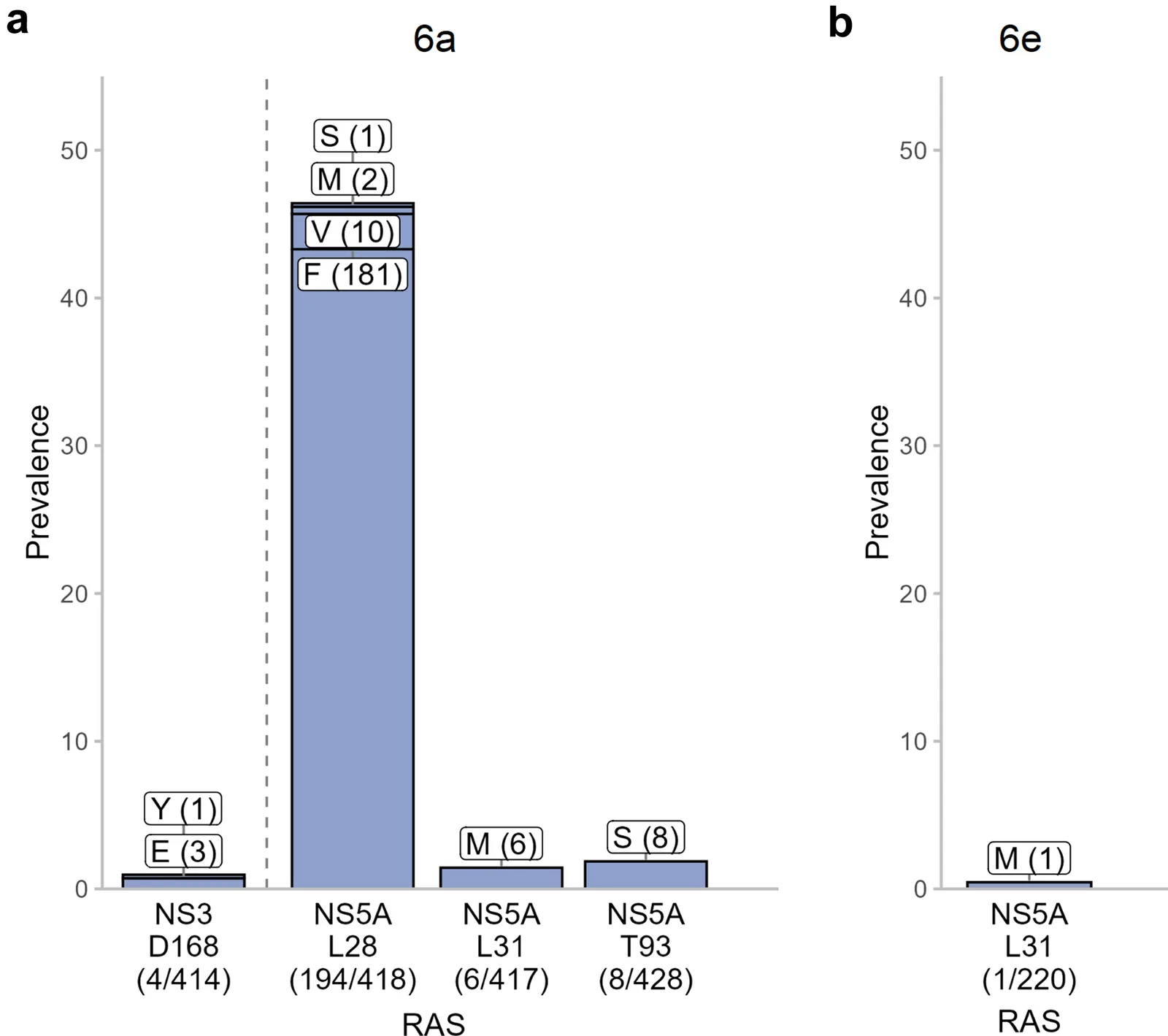
**Prevalence and distribution of clinical RASs in HCV subtypes 6a (a) and 6e (b).** Bar charts illustrate the prevalence of resistance-associated substitutions at specific polymorphic sites within the NS3, NS5A, and NS5B regions. The x-axis labels identify the specific gene and amino acid position, along with the total count and proportion of samples harbouring a mutation. The stacked segments within each bar denote the specific amino acid variants detected; labels indicate the amino acid (single-letter code) and the number of isolates carrying that specific substitution (in parentheses). The dashed vertical line separates RASs located in different viral proteins.

The resistance profile for subtype 6e stood in sharp contrast to 6a. Subtype 6e exhibited an almost complete absence of clinically significant mutations (Fig. 3). The only previously reported clinical RAS detected was NS5A_L31M, which was found in a single sample (0.5%, 1/220). Consequently, the predicted resistance to daclatasvir in subtype 6e was negligible.

#### Subtype 1a

In subtype 1a, the resistance landscape was dominated by the NS3 region. The most common clinical RAS identified was NS3_Q80K, detected in 54.8% (170/310) of sequences. This substitution is associated with substantial resistance to multiple NS3 protease inhibitors, including grazoprevir and voxilaprevir.

**Fig. 4:**
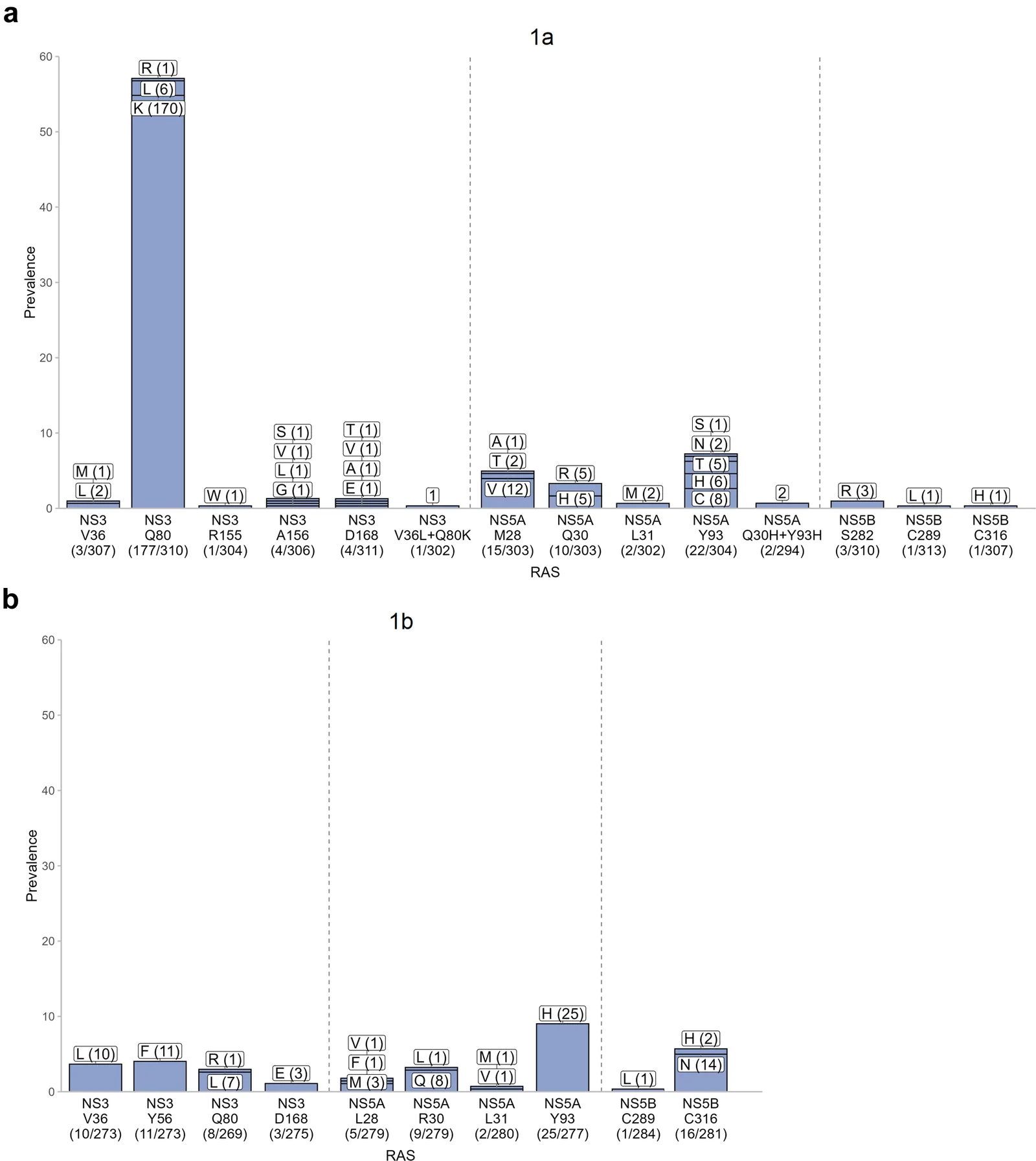
**Prevalence and distribution of clinical RASs in HCV subtypes 1a (a) and 1b (b).** Bar charts illustrate the prevalence of resistance-associated substitutions at known polymorphic sites within the NS3, NS5A, and NS5B regions. The x-axis labels identify the specific gene and amino acid position, along with the total count and proportion of samples harbouring a mutation. The stacked segments within each bar denote the specific amino acid variants detected; labels indicate the amino acid (single-letter code) and the number of isolates carrying that specific substitution (in parentheses). The dashed vertical line separates RASs located in different viral proteins.

In contrast, clinical RASs in the NS5A and NS5B regions were significantly less frequent. In the NS5A region, the most common variation occurred at position 93 (Y93C/H/N/S/T), which was observed in 7.2% (22/304) of samples (Fig. 4). Substitutions at this position are associated with reduced susceptibility to a broad range of NS5A inhibitors, including daclatasvir, ledipasvir, pibrentasvir, elbasvir, and velpatasvir. Resistance-associated mutations in the NS5B region were rare, resulting in a very low prevalence of predicted resistance to sofosbuvir (1.3%, 4/316) (Supplementary Table S4).

Regarding sub-clinical substitutions, most presented at low prevalence (typically ≤1%). However, a few specific polymorphisms were more notable, including NS3_I170V (6.1%, 19/312), NS5A_H58P (5.1%, 16/311), and NS5B_E237G (10.7%, 34/317) (Supplementary Figure S6). These variants have been linked to reduced susceptibility to grazoprevir, daclatasvir, and sofosbuvir, respectively (Supplementary Table S5).

#### Subtype 1b

The resistance profile of subtype 1b differed notably from that of subtype 1a, most significantly by the complete absence of the NS3_Q80K substitution, which was highly prevalent in subtype 1a. Instead, the most common clinical RAS identified in subtype 1b was NS5A_Y93H, detected in 9.0% (25/277) of sequences (Fig. 4). This substitution is a major contributor to resistance against NS5A inhibitors, conferring reduced susceptibility to daclatasvir, elbasvir, ledipasvir, pibrentasvir, and velpatasvir.

Predicted resistance in subtype 1b was more broadly distributed across DAA classes compared to other subtypes. Measurable resistance frequencies were observed for the NS3 inhibitor grazoprevir (11.5%, 32/279), various NS5A inhibitors (ranging from 9.3% to 13.6%), and the NS5B inhibitor sofosbuvir (5.9%, 17/288) (Supplementary Table S4).

Regarding sub-clinical substitutions, most appeared at low prevalence, typically affecting only a small number of patients. A notable exception was the NS5A_F37L substitution, which was observed at a relatively high frequency (18.1%, 51/282) (Supplementary Figure S6). While currently classified as sub-clinical, the high prevalence of this polymorphism suggests it may warrant further investigation.

#### Genotype 2, subtypes 2a and 2m

Genotype 2 subtypes exhibited a resistance profile characterised by a high prevalence of previously reported clinical RASs, which were concentrated almost exclusively in the NS5A region. The widespread presence of several substitutions across these subtypes suggests that many of these variants likely represent subtype-defining polymorphisms for these lineages in Viet Nam rather than acquired resistance mutations selected under prior treatment pressure. While these substitutions may represent naturally occurring subtype-specific polymorphisms, they occur at previously reported resistance-associated positions within the NS5A region.

In subtype 2a, NS5A_L31M and NS5A_Y93N were the most prevalent previously reported clinical RASs, detected in 90.8% (59/65) and 42.4% (28/66) of sequences, respectively. Consistent with the high prevalence of these NS5A substitutions, predicted resistance prevalence was high for key NS5A inhibitors: daclatasvir (93.8%, 61/65), elbasvir (90.8%, 59/65), and velpatasvir (95.4%, 62/65). Additionally, the sub-clinical RAS NS5A_T24A was observed in 6.2% (4/65) of subtype 2a sequences. While its individual frequency was low, this pattern warrants attention as it may further contribute to reduced DAA susceptibility.

**Fig. 5:**
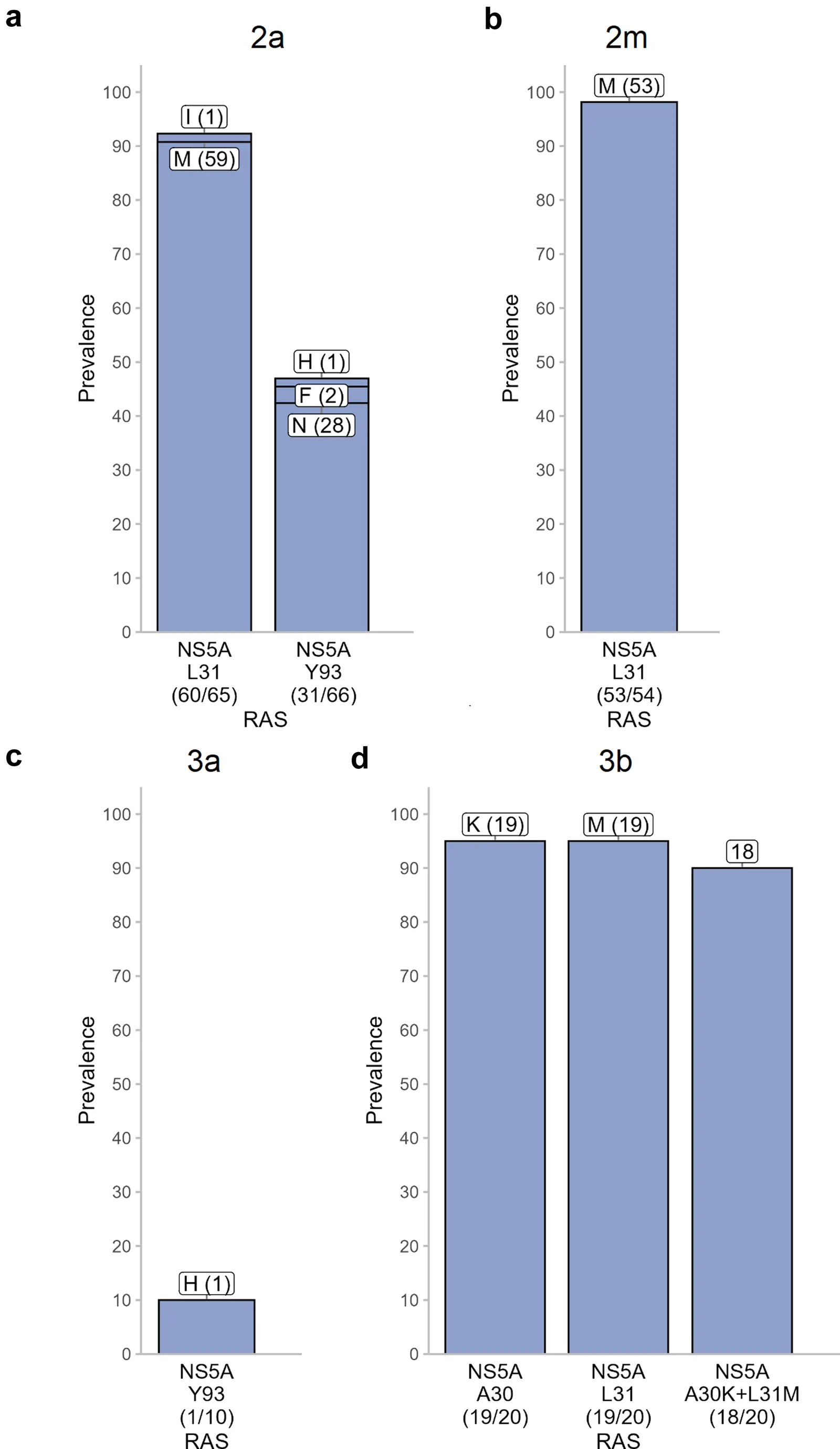
**Prevalence and distribution of clinical RASs in HCV subtypes 2a (a), 2m (b), 3a (c), and 3b (d).** Bar charts illustrate the prevalence of resistance-associated substitutions at known polymorphic sites within the NS3, NS5A, and NS5B regions. The x-axis labels identify the specific gene and amino acid position, along with the total count and proportion of samples harbouring a mutation. The stacked segments within each bar denote the specific amino acid variants detected; labels indicate the amino acid (single-letter code) and the number of isolates carrying that specific substitution (in parentheses). The dashed vertical line separates RASs located in different viral proteins.

Subtype 2m displayed a similarly restricted resistance pattern dominated by NS5A_L31M, which was detected in 98.1% (53/54) of sequences and corresponded to a predicted daclatasvir resistance prevalence of 98.1%. Notably, no clinical RASs were identified in the NS3 or NS5B regions of either genotype 2 subtype, indicating that the predicted resistance profile was confined to NS5A (Fig. 5).

#### Genotype 3, subtypes 3a and 3b

Genotype 3 subtypes displayed strikingly contrasting resistance profiles, highlighting the critical importance of subtype-level discrimination. In subtype 3a, clinically relevant resistance was minimal. NS5A_Y93H was the only previously reported significant clinical RAS detected, appearing in just 10% (1/10) of sequences. While some sub-clinical substitutions were observed such as NS3_A166S (30.0%, 3/10), NS5A_A62L (20.0%, 2/10), NS5B_A150V (40.0%, 4/10), and NS5B_K206E (10.0%, 1/10), the overall profile suggested a population that remains largely susceptible to current DAAs.

In stark contrast, subtype 3b demonstrated a high prevalence of previously reported clinical NS5A RASs. The substitutions NS5A_A30K and NS5A_L31M were each detected in 95.0% (19/20) of sequences, with the dual combination (A30K + L31M) present in 90.0% (18/20) of sequences. The near-fixation of these substitutions indicates they are likely subtype-defining wild-type polymorphisms rather than acquired mutations. Consistent with the predominance of these NS5A substitutions, predicted resistance prevalence reached 100% for velpatasvir among subtype 3b isolates. These observations demonstrate marked differences in the baseline distribution of previously reported clinical NS5A RASs between subtypes 3a and 3b (Fig. 5).

### Impact on direct-acting antivirals

An assessment of subtype-specific resistance-associated substitutions demonstrated marked heterogeneity in predicted resistance profiles across individual DAA classes and HCV subtypes. Among NS3 protease inhibitors, grazoprevir-associated RASs demonstrated marked subtype-specific differences. Subtype 1a demonstrated the highest prevalence (55.6%, 179/322), followed by subtype 1b (11.5%, 32/279). In contrast, subtype 6a demonstrated substantially lower prevalence (0.9%, 4/430). Voxilaprevir-associated RASs similarly demonstrated elevated prevalence in subtype 1a (56.2%, 181/322). Glecaprevir-associated substitutions remained rare across nearly all subtypes, with only 1.6% (5/310) in subtype 1a and 0.2% (1/421) prevalence observed in subtype 6a.

Among NS5A inhibitors, daclatasvir demonstrated the highest overall RAS prevalence, primarily reflecting the high prevalence observed in genotype 2 subtypes and subtype 6a. Subtype 2a demonstrated remarkably high prevalence of daclatasvir-associated RASs (93.8%, 61/65), while subtype 2m demonstrated RASs in 98.1% (53/54) of sequences. Among genotype 6 infections, subtype 6a demonstrated substantially higher daclatasvir-associated RAS prevalence (44.5%, 186/418) compared with subtype 6e, where only 0.5% (1/220) of sequences carried associated substitutions. Within genotype 1 infections, subtype 1a demonstrated slightly lower prevalence of daclatasvir-associated RASs (12.8%, 39/304) compared with subtype 1b (13.6%, 38/280), whereas subtype 3a demonstrated low prevalence (10.0%, 1/10).

Velpatasvir-associated RAS prevalence demonstrated substantial subtype variability. Subtypes 2a and 3b demonstrated the highest prevalence, with substitutions observed in 95.4% (62/65) and 100.0% (20/20) of sequences, respectively. In contrast, subtype 6a demonstrated substantially lower prevalence (6.2%, 26/418), while subtype 6e demonstrated very low prevalence of RASs. Among genotype 1 infections, velpatasvir-associated RAS prevalence remained intermediate in subtype 1a (13.5%, 41/304) and subtype 1b (9.6%, 27/280). Similar trends were observed for ledipasvir, where genotype 1 infections demonstrated intermediate prevalence (13.4%, 41/305 in subtype 1a and 10.7%, 30/280 in subtype 1b), while subtype 3a remained low (10.0%, 1/10). Subtype 6a demonstrated only 1.4% (6/418) prevalence of elbasvir-associated RASs, whereas subtype 6e demonstrated no detected elbasvir-associated substitutions. Intermediate prevalence of elbasvir-associated RASs was observed in subtype 1a (13.1%, 40/305) and subtype 1b (12.5%, 35/280), while subtype 3a demonstrated low prevalence (10.0%, 1/10). In contrast, subtype 2a demonstrated markedly higher prevalence of elbasvir-associated RASs (90.8%, 59/65).

Pibrentasvir-associated RASs remained uncommon across most subtypes and demonstrated the lowest prevalence among evaluated NS5A inhibitors. Only 2.8% (45/1584) of sequences carried pibrentasvir-associated substitutions overall (Supplementary Figure S7). Among genotype 1 infections, subtype 1a demonstrated 5.9% (18/304) prevalence, while subtype 1b demonstrated 9.3% (26/280) and low prevalence in subtype 3a (10.0%, 1/10).

Sofosbuvir demonstrated a high overall genetic barrier to resistance, with associated substitutions identified in only 1.3% (21/1576) of sequences overall. Among genotype 1 infections, subtype 1a demonstrated 1.3% (4/316) prevalence, while subtype 1b demonstrated 5.9% (17/288). No sofosbuvir-associated substitutions were detected in genotype 2, genotype 3, or genotype 6 subtypes included in this analysis.

These findings demonstrate substantial subtype-specific differences in baseline RAS prevalence across currently used DAA classes. While several subtypes demonstrated low prevalence across most DAAs, markedly elevated prevalence was observed in genotype 2 subtypes and subtype 3b, particularly for NS5A inhibitors. In contrast, glecaprevir, pibrentasvir, and sofosbuvir generally retained low overall RAS prevalence across most circulating subtypes within the Vietnamese cohort (Supplementary Figure S8).

### Subtype-specific prevalence of resistance-associated substitutions across DAA regimens

**Fig. 6:**
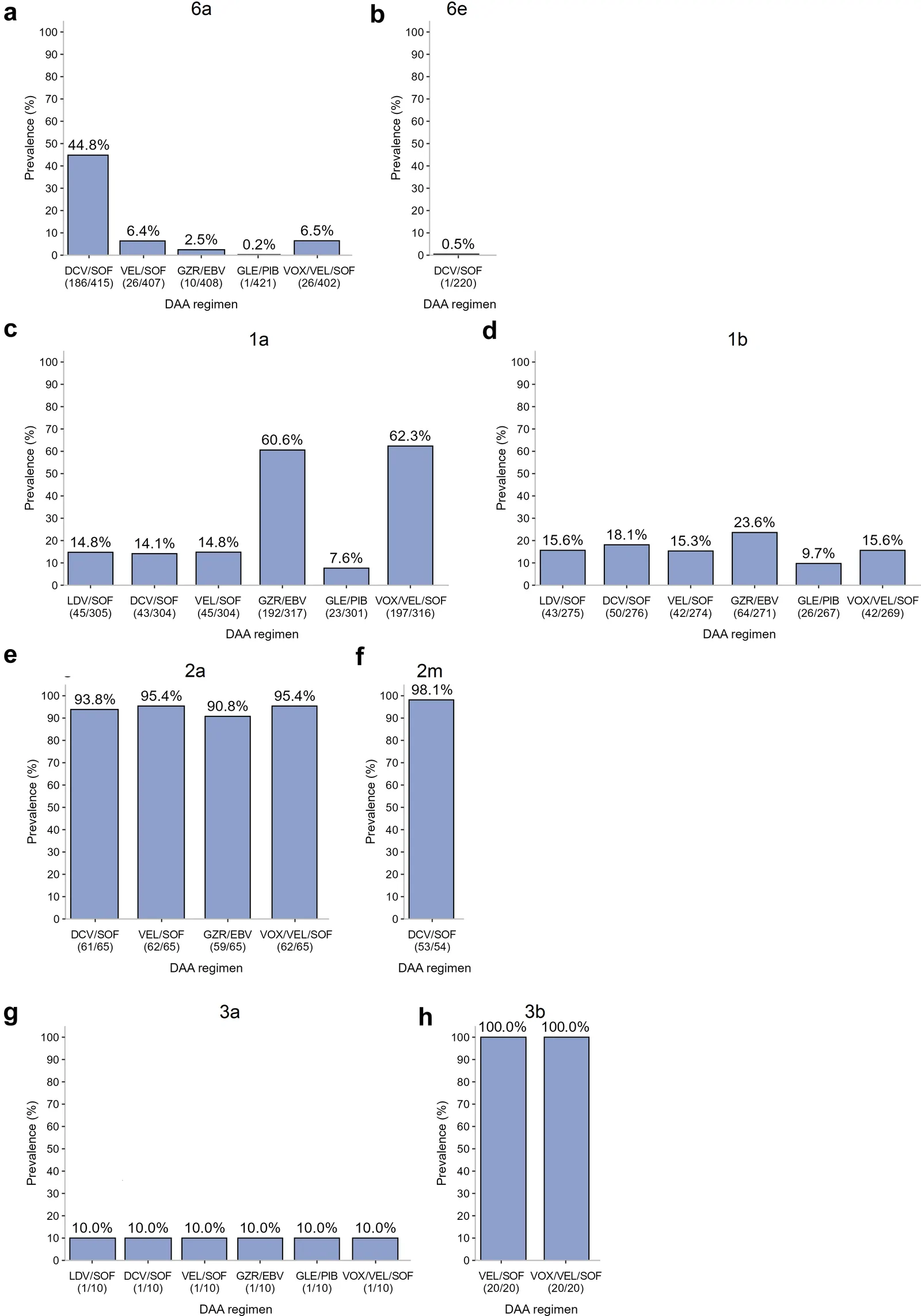
**Prevalence of regimen-level resistance-associated substitutions across HCV subtypes.** Bar plots illustrate the prevalence of RASs affecting currently used direct-acting antiviral regimens among HCV subtypes identified in the Vietnamese cohort. Panels represent subtype-specific analyses for subtypes 6a (a), 6e (b), 1a (c), 1b (d), 2a (e), 2m (f), 3a (g), and 3b (h). Evaluated regimens include ledipasvir/sofosbuvir (LDV/SOF), daclatasvir/sofosbuvir (DCV/SOF), velpatasvir/sofosbuvir (VEL/SOF), grazoprevir/elbasvir (GZR/EBV), glecaprevir/pibrentasvir (GLE/PIB), and voxilaprevir/velpatasvir/sofosbuvir (VOX/VEL/SOF). Percentages above bars represent prevalence of RASs, while values beneath each bar indicate the number of sequences over the total number of sequences analysed for each subtype–regimen combination.

Regimen-level analysis of baseline resistance-associated substitutions demonstrated substantial variability in RAS prevalence across HCV subtypes and currently used DAA combinations (Fig. 6). Among genotype 6 subtypes, subtype 6a showed the highest prevalence of RASs affecting daclatasvir/sofosbuvir (44.8%, 186/415), while substantially lower prevalence was observed for velpatasvir/sofosbuvir (6.4%, 26/407), grazoprevir/elbasvir (2.5%, 10/408), glecaprevir/pibrentasvir (0.2%, 1/421), and voxilaprevir/velpatasvir/sofosbuvir (6.5%, 26/402). In contrast, subtype 6e demonstrated minimal regimen-level RAS prevalence, with only 0.5% (1/220) of sequences carrying RASs associated with daclatasvir/sofosbuvir.

Within genotype 1, subtype 1a demonstrated markedly higher prevalence of RASs affecting grazoprevir/elbasvir (60.6%, 192/317) and voxilaprevir/velpatasvir/sofosbuvir (62.3%, 197/316), while intermediate prevalence was observed for ledipasvir/sofosbuvir (14.8%, 45/305), daclatasvir/sofosbuvir (14.1%, 43/304), and velpatasvir/sofosbuvir (14.8%, 45/304). Glecaprevir/pibrentasvir demonstrated the lowest RAS prevalence in subtype 1a (7.6%, 23/301). Similarly, subtype 1b demonstrated overall lower regimen-level RAS prevalence compared with subtype 1a, with glecaprevir/pibrentasvir again showing the lowest prevalence (9.7%, 26/267). Among the remaining regimens, daclatasvir/sofosbuvir (18.1%, 50/276) demonstrated slightly higher RAS prevalence than ledipasvir/sofosbuvir (15.6%, 43/275) and velpatasvir/sofosbuvir (15.3%, 42/274) within subtype 1b.

In contrast, genotype 2 and subtype 3b demonstrated consistently high regimen-level RAS prevalence. Subtype 2a demonstrated RAS prevalence exceeding 90% across all evaluated regimens, including daclatasvir/sofosbuvir (93.8%, 61/65), velpatasvir/sofosbuvir (95.4%, 62/65), grazoprevir/elbasvir (90.8%, 59/65), and voxilaprevir/velpatasvir/sofosbuvir (95.4%, 62/65). Similarly, subtype 2m demonstrated 98.1% (53/54) RAS prevalence affecting daclatasvir/sofosbuvir. Subtype 3b demonstrated RAS prevalence for both velpatasvir/sofosbuvir and voxilaprevir/velpatasvir/sofosbuvir (100.0%, 20/20). However, subtype 3a demonstrated substantially lower prevalence, with RASs affecting each evaluated regimens observed in only 10.0% (1/10) of sequences.

### Emerging concerns: putative novel RASs

Beyond established resistance mutations, we identified several putative new RAS variants at positions known to confer resistance in other HCV subtypes but not yet characterised in the subtypes under investigation.

In genotypes 2 and 3, high-frequency substitutions were observed in the NS3 and NS5A regions. In subtype 2a, NS3_36L was found in 97.1% (67/69) of sequences and NS5A_30K in 90.8% (59/65). In subtype 2m, the NS3_36L was universally present in 100% (56/56) of sequences, while NS3_56F was detected in 77.6% (45/58) of sequences. Additionally, subtype 2m sequences frequently harboured substitutions at NS5A position 30, specifically 30R (79.6%, 43/54) and 30K (20.4%, 11/54). Similarly, the NS3_36L substitution was fixed (100.0%) in all subtype 3a (10/10) and subtype 3b (20/20) sequences, with subtype 3b also universally carrying NS5A_28M (100.0%, 20/20).

Given that resistance profiles for genotype 6 remain understudied, we identified numerous putative RASs across its diverse subtypes. In subtype 6a, NS3_80K, a clinically significant mutation in genotype 1a, was detected in 96.0% (403/420) of sequences, while NS5B_289L was found in 3.0% (13/433). Subtype 6e exhibited a complex array of novel variants. At NS5A position 28, variants 28V and 28M were found in 57.3% (125/218) and 41.3% (90/218) of subtype 6e sequences, respectively. The substitution NS5A_30S was detected in 97.7% (213/218) of sequences. At position 93, NS5A_93T was dominant (96.0%, 217/226), with 93S appearing less frequently (2.7%, 6/226). Furthermore, NS5B_289L was nearly universal in subtype 6e (99.6%, 225/226).

High-frequency putative RASs were also conserved in other genotype 6 subtypes. In subtype 6h, NS5A_28V was present in 100% (37/37) of sequences, and NS5A_93T in 94.4% (34/36). In subtype 6l, substitutions NS5A_28V (85.0%, 34/40), NS5A_93T (97.5%, 39/40), and NS5B_289L (97.5%, 39/40) were highly prevalent. Finally, subtype 6o displayed novel variants including NS5A_93S (70.0%, 7/10) and NS5B_289L observed in 100% (11/11) of sequences.

The high prevalence and in many instances, fixation of these substitutions suggests that they represent subtype-defining wild-type polymorphisms rather than acquired mutations driven by selective drug pressure. Nevertheless, because these substitutions occur at positions previously implicated in DAA resistance in other HCV subtypes, they represent important candidates for future phenotypic evaluation. Their high prevalence highlights the need for continued molecular surveillance, particularly among understudied genotype 6 subtypes, to determine whether these conserved polymorphisms contribute to altered antiviral susceptibility or influence treatment response (Supplementary Figure S9).

## Discussion

This study represents one of the most comprehensive genomic characterisations of Hepatitis C virus in Viet Nam to date. By analysing whole-genome sequences from a large, geographically diverse cohort, we have provided a high-resolution overview of the country's complex HCV epidemic. Our findings confirm the unique predominance of genotype 6 and demonstrate substantial subtype-specific variability in baseline RAS prevalence across currently used DAA classes and regimens. In particular, several genotype 2 and subtype 3b lineages carried naturally occurring subtype-defining polymorphisms at previously reported resistance-associated positions, predominantly within the NS5A region. Although the clinical significance of these substitutions requires cautious interpretation in the absence of direct phenotypic validation, the observed subtype-specific RAS patterns support continued genomic surveillance and may inform the selection of DAA regimens where subtype information and resistance testing are available, particularly in regions with highly diverse HCV populations.

Our results confirm that the Vietnamese HCV epidemic is characterised by high genetic diversity, driven by the co-circulation of four major genotypes. Consistent with regional data, genotype 6 remains the dominant strain nationally (50.3%), particularly in the southern and northern regions, while genotype 1 predominates in central Viet Nam; together, genotypes 6 and 1 account for almost 90% of infections. Additionally, genotype 2 was almost exclusively concentrated in the south, and genotype 3 displayed distinct regional clustering in the north. This strong association between regions and HCV subtypes suggests that diverse transmission pathways and historical founder effects play a crucial role in viral spread.

The predominance of subtype 2m within genotype 2 represents an intriguing epidemiological and evolutionary observation in the Vietnamese HCV epidemic. Geographic analyses demonstrated that genotype 2 infections were concentrated predominantly within southern Viet Nam, particularly the Mekong River Delta, where subtype 2m represented the dominant genotype 2 lineage. Previous phylogeographic studies have suggested that several genotype 2 subtypes, including 2i, 2j, and 2m, may have been introduced into Viet Nam during historical population movements and colonial trade periods.^2^^,^^30^^,^^31^ The observed concentration of subtype 2m within the Mekong River Delta is compatible with this hypothesis and may reflect subsequent local expansion within southern Vietnamese transmission networks. However, the absence of extensive regional comparator sequences from neighbouring Southeast Asian countries and the limited number of globally sampled subtype 2m sequences substantially restrict phylogeographic resolution. Therefore, while the current findings support long-term endemic circulation of subtype 2m within southern Viet Nam, the precise geographic origin and introduction pathway of this lineage remain uncertain.

We observed significant demographic partitioning among genotypes that likely reflects distinct transmission dynamics. Genotype 3 was strongly associated with younger males (median age 36), potentially indicating more recent transmission networks, whereas genotype 2 was concentrated in older females (median age 56), potentially reflecting differences in historical exposure patterns or transmission networks. Furthermore, the high prevalence of genotypes 1 and 2 among people living with HIV could indicate a link between these genotypes and high-risk behaviours, such as injection drug use, which has been well-documented in Viet Nam.^12^ These associations are critical for public health targeting; for instance, screening programmes targeting younger populations may encounter different viral profiles than those screening older, general populations.

A pivotal finding of this study is the high prevalence of pre-existing clinical RASs in genotypes 2 and 3. We observed that certain clinical RASs in the NS5A region, specifically L31M in genotype 2 and the dual A30K + L31M mutation in subtype 3b, were fixed in nearly 100% of sequences. The ubiquity of these substitutions suggests they are not acquired mutations selected by prior therapy, but rather subtype-defining wild-type polymorphisms.

These naturally occurring subtype-specific polymorphism patterns may have important clinical implications. For subtype 3b, these polymorphisms were associated with a predicted resistance prevalence of 100% to velpatasvir, a frontline NS5A inhibitor. Similarly, genotype 2 subtypes showed more than 90% predicted resistance to daclatasvir, elbasvir and velpatasvir. These findings indicate that genotype 2 subtypes and subtype 3b carry subtype-defining polymorphisms at previously reported resistance-associated positions that correspond to high predicted resistance prevalence for specific DAAs. Consequently, these subtype-specific polymorphism patterns may warrant consideration in clinical settings where NS5A-based therapies are used without resistance testing, particularly in subgroups previously associated with increased risk of virologic failure.^18^^,^^32^, ^33^, ^34^

Given that genotype 6 is largely neglected in global clinical trials, our detailed characterisation of its subtypes and their RAS profile fills a critical knowledge gap. While genotype 6 had the lowest overall clinical RAS prevalence (25.1%), this aggregate figure masks significant subtype-specific risks. In subtype 6a, the most common subtype in Viet Nam, the NS5A_L28F substitution was dominant and we observed a predicted daclatasvir resistance prevalence of 44.5%. Conversely, subtype 6e demonstrated a low prevalence of previously reported clinical RASs.

Furthermore, we identified numerous “putative new RASs” in genotype 6, such as NS3_80K in subtype 6a; NS5A_28V/M, 30S, 93T and NS5B_289L in subtype 6e, which appear to be natural polymorphisms. While their phenotypic impact remains to be fully characterised, their location at critical drug-binding sites identifies them as important candidates for future phenotypic evaluation. This highlights the large genetic diversity of HCV and therefore HCV resistance and the necessity of looking beyond the well-characterised genotype 1 RASs when managing non-1 genotypes.

The findings of this study are relevant for HCV management in Viet Nam where sofosbuvir-based regimens have been widely used, particularly sofosbuvir/daclatasvir, sofosbuvir/ledipasvir, and more recently sofosbuvir/velpatasvir. Previous Vietnamese studies have shown high SVR rates with sofosbuvir-based therapy, including genotype 6 infections, although some studies suggested that genotype 6 may respond slightly less well than genotype 1 in specific real-world cohorts.^35^ Importantly, several clinical studies have demonstrated that high SVR rates can still be achieved despite the presence of baseline RASs, indicating that naturally occurring substitutions alone are often insufficient to predict treatment failure. The low prevalence of NS5B-associated RASs further supports the retained activity of sofosbuvir-containing regimens across the majority of circulating HCV lineages identified in Viet Nam.

In contrast, substantial subtype-specific variability was observed in baseline NS5A-associated RAS prevalence. The highest prevalence of regimen-level RASs was observed for daclatasvir- and velpatasvir-containing regimens among several subtypes, particularly genotype 2 lineages and subtype 3b, where naturally occurring polymorphisms at recognised resistance-associated positions were common. Subtype 6a also demonstrated elevated RAS prevalence for daclatasvir-containing regimens compared with other genotype 6 subtypes. These findings suggest that the distribution of naturally occurring NS5A polymorphisms differs markedly between HCV subtypes and may influence the resistance landscape of specific NS5A inhibitors. In contrast, glecaprevir/pibrentasvir demonstrated comparatively low RAS prevalence across several major circulating subtypes, including 1a, 1b, 3a, and 6a, consistent with the high genetic barrier associated with pibrentasvir-containing regimens and the strong clinical efficacy reported for diverse HCV genotypes, including genotype 6 infections.^36^ Although these observations are based on sequence-defined RASs rather than phenotypic validation, they highlight the importance of continued resistance surveillance, particularly among patients experiencing DAA treatment failure.

Together, these findings support continued use of effective pan-genotypic DAA regimens in Viet Nam. Baseline RAS testing is unlikely to be either necessary or affordable for all patients. Instead, targeted RAS assessment may be more informative in selected clinical settings, including patients with prior DAA treatment failure, infections involving genotype 2 or subtype 3b, cirrhotic patients requiring retreatment, or cases where optimisation of therapy is clinically important.

There are several limitations to this study. First, the cohort was derived from six clinical research studies conducted over a 10-year period rather than from population-based sampling, which may introduce heterogeneity in patient selection, enrolment criteria, and clinical characteristics. In addition, the majority of enrolled participants originated from sites in southern Viet Nam, particularly Ho Chi Minh City, resulting in geographical imbalance that may have influenced the observed prevalence of certain genotypes and subtypes, particularly genotype 3. Furthermore, because the cohort combined participants from multiple independent studies conducted across different time periods, geographic regions, and recruitment frameworks, the dataset was not designed to reliably infer temporal trends in HCV genotype distribution at the national level. Consequently, apparent temporal differences may reflect variation in study enrolment strategies and underlying patient populations rather than true longitudinal epidemiological changes within Viet Nam. Nevertheless, the overall genotype distribution observed in this cohort remained consistent with previous epidemiological studies from Viet Nam, in which genotype 6 predominated, followed by genotypes 1, 2, and 3.^11^^,^^37^ Sensitivity analyses excluding participants from the VIETNARMS trial, which included targeted enrolment of additional genotype 6 infections, demonstrated similar overall epidemiological patterns to those observed in the full cohort.

Second, the resistance interpretations presented in this manuscript were based primarily on previously reported resistance-associated positions, literature-based classifications, and in silico genomic inference, without direct phenotypic validation. Consequently, the functional and clinical significance of several observed substitutions, particularly high-frequency subtype-specific polymorphisms and putative new RASs, should be interpreted cautiously. Many of these substitutions may represent naturally occurring subtype-defining consensus polymorphisms rather than confirmed resistance determinants. This limitation is particularly relevant for understudied genotype 6 subtypes, where phenotypic resistance data remain limited.

Finally, the current study focused primarily on the genomic characterisation and subtype-specific prevalence of naturally occurring baseline RASs and did not include direct treatment outcome analyses. Future studies incorporating phenotypic resistance assays together with longitudinal treatment outcome data will therefore be essential to determine the functional and clinical relevance of these subtype-specific polymorphism patterns within diverse HCV populations.

In conclusion, the HCV epidemic in Viet Nam is defined by significant genetic diversity and a high burden of subtype-specific baseline resistance-associated polymorphisms in specific subtypes. As Viet Nam moves towards HCV elimination, integrating subtype-level surveillance and RAS testing into national strategies in selected clinical settings may support treatment optimisation and prevent the expansion of resistant viral populations.

## Contributors

MAA, EB, SP, GSC, MR, ASW conceived and designed the study. CLN and MAA wrote the first draft of the manuscript. CLN, GA, TD, DJ, FX conducted laboratory testing. MAA, CLN, HC had full access to all study data. MAA and CLN verified the underlying data. CLN, HC, MAA carried out data analysis. BF, MMM, LM, HLM, NVVC, TDT, CLN, TPN, HVTT, GET, HRvD, JD, EK, SP, EB, TLV contributed to data acquisition in the field, and edited the manuscript. GSC and MAA had final responsibility for the decision to submit the manuscript for publication. All authors read and approved the final manuscript.

## Data sharing statement

The data generated and analysed in this study will be made available in accordance with the Oxford University Clinical Research Unit (OUCRU) data sharing policy, which operates under a controlled-access framework to protect participant confidentiality and ongoing research activities. De-identified individual participant data, accompanying metadata, and data dictionaries underlying the findings of this study will be available following publication, subject to approval by the OUCRU Data Access Committee. Requests for access should be submitted to dac@oucru.org. Applicants will be required to complete a data access request form (available at https://www.oucru.org/data-sharing-policy/) and sign a data access agreement.

## Editor note

The Lancet Group takes a neutral position with respect to territorial claims in published maps and institutional affiliations.

## Declaration of interests

SP reports grants paid to her institution, University College London, in support of trials, from EDCTP-2, EDCTP-3, the Medical Research Council (MRC), the National Institute for Health and Care Research (NIHR), the National Health and Medical Research Council (NHMRC), Gilead Sciences, ViiV Healthcare, Janssen-Cilag, the National Institutes of Health (NIH), and the European Commission, all unrelated to the present work. SP is Chair of the UK Data Safety Monitoring Board for the IMPEDE-PKD and serves as a member of the UK Data Safety Monitoring Board for the TIPAL trial. All other authors declare no competing interests.

## References

[1] World Health Organization (WHO) (2022).

[2] Le Ngoc C., Tran Thi Thanh T., Tran Thi Lan P. (2019). Differential prevalence and geographic distribution of hepatitis C virus genotypes in acute and chronic hepatitis C patients in Vietnam. PLoS One.

[3] Blach S., Zeuzem S., Manns M. (2017). Global prevalence and genotype distribution of hepatitis C virus infection in 2015: a modelling study. Lancet Gastroenterol Hepatol.

[4] Roudot-Thoraval F. (2021). Epidemiology of hepatitis C virus infection. Clin Res Hepatol Gastroenterol.

[5] Vo-Quang E., Pawlotsky J.M. (2024). “Unusual” HCV genotype subtypes: origin, distribution, sensitivity to direct-acting antiviral drugs and behaviour on antiviral treatment and retreatment. Gut.

[6] Messina J.P., Humphreys I., Flaxman A. (2015). Global distribution and prevalence of hepatitis C virus genotypes. Hepatology.

[7] Poovorawan Y. (2012). Epidemiology of hepatitis C virus - genotypes 1 through 6 in Asia and the world. Int J Infect Dis.

[8] Irekeola A.A., Malek N.A., Wada Y., Mustaffa N., Muhamad N.I., Shueb R.H. (2021). Prevalence of HCV genotypes and subtypes in Southeast Asia: a systematic review and meta-analysis. PLoS One.

[9] Zumaeta Villena E. (2006). Transmission routes of hepatitis C virus infection. Ann Hepatol.

[10] Flower B., Du Hong D., Vu Thi Kim H. (2022). Seroprevalence of Hepatitis B, C and D in Vietnam: a systematic review and meta-analysis. Lancet Reg Health West Pac.

[11] Pham V.H., Nguyen H.D.P., Ho P.T. (2011). Very high prevalence of hepatitis C virus genotype 6 variants in southern Vietnam: large-scale survey based on sequence determination. Jpn J Infect Dis.

[12] Dunford L., Carr M.J., Dean J. (2012). Hepatitis C virus in Vietnam: high prevalence of infection in dialysis and multi-transfused patients involving diverse and novel virus variants. PLoS One.

[13] Zhang Z., Yao Y., Wu W. (2013). Hepatitis C virus genotype diversity among intravenous drug users in Yunnan Province, Southwestern China. PLoS One.

[14] Thi Ngoc P.N., My N.N., Rasheed S. (2023). Public healthcare system utilization for chronic hepatitis C infection in Vietnam. BMC Infect Dis.

[15] Itakura J., Kurosaki M., Kakizaki S. (2020). Features of resistance-associated substitutions after failure of multiple direct-acting antiviral regimens for hepatitis C. JHEP Rep.

[16] Parczewski M., Janczewska E., Pisula A. (2021). HCV resistance-associated substitutions following direct-acting antiviral therapy failure – Real-life data from Poland. Infect Genet Evol.

[17] Mawatari S., Oda K., Tabu K. (2018). The co-existence of NS5A and NS5B resistance-associated substitutions is associated with virologic failure in Hepatitis C Virus genotype 1 patients treated with sofosbuvir and ledipasvir. PLoS One.

[18] Liu Z., Mao X., Wu J. (2021). World-wide prevalence of substitutions in HCV genome associated with resistance to direct-acting antiviral agents. Clin Gastroenterol Hepatol.

[19] Nguyen T.K., Van Le D. (2022). Identification of NS5B resistance-associated mutations in hepatitis C virus circulating in treatment Naïve Vietnamese patients. Infect Drug Resist.

[20] Bunchorntavakul C., Chavalitdhamrong D., Tanwandee T. (2013). Hepatitis c genotype 6: a concise review and response-guided therapy proposal. World J Hepatol.

[21] Gaidatzis D., Lerch A., Hahne F., Stadler M.B. (2015). QuasR: quantification and annotation of short reads in R. Bioinformatics.

[22] Martin M. (2011). Cutadapt removes adapter sequences from high-throughput sequencing reads. EMBnet J.

[23] Illumina (2012). Illumina adapter sequences: oligonucleotides for TruSeq, Nextera, and index kits. http://www.illumina.com.

[24] Nurk S., Koren S., Rhie A. (2022). The complete sequence of a human genome. Science.

[25] Langmead B., Salzberg S.L. (2012). Fast gapped-read alignment with Bowtie 2. Nat Methods.

[26] Li H., Durbin R. (2009). Fast and accurate short read alignment with Burrows–Wheeler transform. Bioinformatics.

[27] Yang X., Charlebois P., Gnerre S. (2012). De novo assembly of highly diverse viral populations. BMC Genomics.

[28] Lefkowitz E.J., Dempsey D.M., Hendrickson R.C., Orton R.J., Siddell S.G., Smith D.B. (2018). Virus taxonomy: the database of the International Committee on Taxonomy of Viruses (ICTV). Nucleic Acids Res.

[29] Wymant C., Blanquart F., Golubchik T. (2018). Easy and accurate reconstruction of whole HIV genomes from short-read sequence data with shiver. Virus Evol.

[30] Li C., Yuan M., Lu L. (2014). The genetic diversity and evolutionary history of hepatitis C virus in Vietnam. Virology.

[31] Markov P.V., van de Laar T.J., Thomas X.V. (2012). Colonial history and contemporary transmission shape the genetic diversity of hepatitis C virus genotype 2 in Amsterdam. J Virol.

[32] Bertoli A., Sorbo M.C., Aragri M. (2018). Prevalence of single and multiple natural NS3, NS5A and NS5B resistance-associated substitutions in hepatitis C virus genotypes 1-4 in Italy. Sci Rep.

[33] Wyles D.L., Luetkemeyer A.F. (2017). Understanding hepatitis C virus drug resistance: clinical implications for current and future regimens. Top Antivir Med.

[34] Simicic P., Grgic I., Santak M., Vince A., Lepej S.Z. (2019). Frequency of baseline NS5A resistance-associated substitutions in patients infected with genotype 1 of hepatitis C virus in Croatia. Microb Pathog.

[35] Flower B., McCabe L., Le Ngoc C. (2021). High cure rates for hepatitis C virus genotype 6 in advanced liver fibrosis with 12 weeks Sofosbuvir and Daclatasvir: the Vietnam SEARCH study. Open Forum Infect Dis.

[36] Asselah T., Lee S.S., Yao B.B. (2019). Efficacy and safety of glecaprevir/pibrentasvir in patients with chronic hepatitis C virus genotype 5 or 6 infection (ENDURANCE-5,6): an open-label, multicentre, phase 3b trial. Lancet Gastroenterol Hepatol.

[37] Duc A.P., Leuangwutiwong P., Jittmittraphap A. (2009). High prevalence of hepatitis C virus genotype 6 in Vietnam. Asian Pac J Allergy Immunol.

