# Supplementary material for: Unravelling HCV diversity and resistance in Viet Nam: a cross-sectional analysis
Source: Lancet Reg Health West Pac. 2026 Aug 19;73:101956. doi: 10.1016/j.lanwpc.2026.101956 (PMC13520911; doi:10.1016/j.lanwpc.2026.101956)
Supplement: Supplementary file 1 [file mmc1.docx]

**Supplementary Figure 1.** **Reference list of clinically relevant RAS positions in NS3, NS5A, and NS5B genes.** This displays key RAS positions within the HCV NS3, NS5A, and NS5B regions. These positions have been well-established in international HCV treatment guidelines or reported in clinical case studies to confer reduced susceptibility to DAAs. This reference was used to identify and classified important RASs in the analysis of Vietnam HCV isolates.

***Supplementary Figure 2. Phylogenetic placement of 1,649 HCV isolates from Viet Nam within a global HCV reference framework.*** *A maximum likelihood phylogenetic tree constructed from whole-genome HCV sequences illustrates the evolutionary relationships between Vietnamese HCV isolates and global reference sequences representing all eight recognized HCV genotypes and their known subtypes. Sequences generated in the present study are highlighted according to genotype: genotype 1 (yellow), genotype 2 (green), genotype 3 (blue), and genotype 6 (red), whereas global reference sequences are shown in grey. The outer ring indicates genotype assignment, and major subtype clades are labelled along the circumference of the tree.*

***Supplementary Figure 3.*** ***Phylogenetic analysis of 1,649 HCV isolates from Viet Nam.*** *A maximum likelihood tree constructed from whole-genome sequences illustrates the genetic diversity of the cohort. Branches are coloured by genotype: genotype 1 (yellow), genotype 2 (green), genotype 3 (blue), and genotype 6 (red). Major clades corresponding to specific subtypes are labelled at the terminal branches. The scale bar represents 0.2 nucleotide substitutions per site.*

***Supplementary Figure 4. Geographic distribution of HCV genotypes in Viet Nam.*** *(a) Regional distribution of HCV genotypes. Pie charts represent the proportions of genotypes 1 (yellow), 2 (green), 3 (blue), and 6 (red) among sequences obtained from northern (n=273), central (n=106), and southern (n=1,270) Viet Nam. (b–c) Geographical distribution of HCV genotypes 2 (b) and 3 (c) across Viet Nam. Provinces are colour-coded based on the prevalence of the specified genotype (light to dark shading indicating proportions of 1–10%, >10–30%, and >30–50%). The numbers displayed on the maps indicate the count of isolates of that specific genotype identified in each province.*

***Supplementary Figure 5. Geographical distribution of HCV genotypes 1 (a) and 6 (b) across Viet Nam.*** *Each province are shaded according to the number of enrolled patients.*

***Supplementary Figure 6.*** ***Prevalence of sub-clinical RASs associated with resistance to NS3 protease inhibitors, NS5A inhibitors, and NS5B polymerase inhibitors across HCV subtypes detected in Vietnam.*** *Bar charts show the proportion of sequences harbouring mutations at specific amino-acid positions. Labels above the bars indicate the substituted amino acid and the number of isolates carrying that substitution, while counts below the x-axis indicate the number of sequences with the mutation relative to the total sequences analysed. The dashed vertical line separates RASs located in different viral proteins.*

**Supplementary Figure 7.** **Prevalence of resistance to DAAs in general Viet Nam HCV cohort.** Each bar represents the prevalence of patients whose virus harboured at least one RAS associated with resistance to a specific DAA within the drug class: NS3 protease inhibitors (a), NS5A inhibitors (b) and NS5B polymerase inhibitors (c).

***Supplementary Figure 8. Prevalence of predicted resistance to specific DAAs stratified by HCV subtype.*** *Bar charts illustrate the proportion of isolates harbouring at least one clinical RAS associated with resistance to individual direct-acting antivirals. Panels are organised by subtype to highlight distinct resistance profiles. The percentages above the bars indicate the prevalence of predicted resistance, while raw counts (number of resistant sequences / total sequences analysed) are provided below the x-axis labels. Dashed vertical lines separate DAAs according to their viral protein targets.* ***Abbreviations:*** *GLE - glecaprevir; GZR - grazoprevir; VOX - voxilaprevir; DCV - daclatasvir; EBV - elbasvir; LDV - ledipasvir; PIB - pibrentasvir; VEL - velpatasvir; SOF - sofosbuvir.*

**Supplementary Figure 9.** **Schematic representation of putative RASs that were known RASs in other subtypes but newly observed in the examined subtypes in Vietnam isolates.** Each horizontal line represents a HCV subtype (y-axis), with markers showing amino acid substitutions at specific protein positions within each protein (x-axis).

***Supplementary Table 1.*** ***Geographic distribution of HCV genotypes among patients.*** *Values represent the number and proportion of cases within each sub-region.*

| **Genotype** | **Region** | **Sub region** | **n (%)** |
| --- | --- | --- | --- |
| **1** | Northern Vietnam | North Mountains and Midlands | 35 (38%) |
|  |  | Red River Delta | 72 (39.8%) |
|  | Central Vietnam | North Central Coast | 13 (59.1%) |
|  |  | Central Highlands | 39 (46.4%) |
|  | Southern Vietnam | South East | 365 (46.9%) |
|  |  | Mekong River Delta | 125 (25.5%) |
| **2** | Central Vietnam | North Central Coast | 1 (4.5%) |
|  |  | Central Highlands | 2 (2.4%) |
|  | Southern Vietnam | South East | 57 (7.3%) |
|  |  | Mekong River Delta | 80 (16.3%) |
| **3** | Northern Vietnam | North Mountains and Midlands | 8 (8.7%) |
|  |  | Red River Delta | 11 (6.1%) |
|  | Central Vietnam | Central Highlands | 5 (6%) |
|  | Southern Vietnam | South East | 5 (0.6%) |
|  |  | Mekong River Delta | 2 (0.4%) |
| **6** | Northern Vietnam | North Mountains and Midlands | 49 (53.3%) |
|  |  | Red River Delta | 98 (54.1%) |
|  | Central Vietnam | North Central Coast | 8 (36.4%) |
|  |  | Central Highlands | 38 (45.2%) |
|  | Southern Vietnam | South East | 352 (45.2%) |
|  |  | Mekong River Delta | 284 (57.8%) |

***Supplementary Table 2. Geographic distribution of HCV subtypes among patients.*** *Values represent the number and proportion of cases within each sub-region.*

| **Genotype** | **Subtype** | **Region** | **Sub region** | **n (%)** |
| --- | --- | --- | --- | --- |
| **1** | **1a** | Northern Vietnam | North Mountains and Midlands | 21 (22.8%) |
|  |  |  | Red River Delta | 37 (20.4%) |
|  |  | Central Vietnam | North Central Coast | 8 (36.4%) |
|  |  |  | Central Highlands | 18 (21.4%) |
|  |  | Southern Vietnam | South East | 194 (24.9%) |
|  |  |  | Mekong River Delta | 60 (12.2%) |
|  | **1b** | Northern Vietnam | North Mountains and Midlands | 14 (15.2%) |
|  |  |  | Red River Delta | 35 (19.3%) |
|  |  | Central Vietnam | North Central Coast | 5 (22.7%) |
|  |  |  | Central Highlands | 21 (25%) |
|  |  | Southern Vietnam | South East | 171 (22%) |
|  |  |  | Mekong River Delta | 65 (13.2%) |
| **2** | **2a** | Central Vietnam | North Central Coast | 1 (4.5%) |
|  |  |  | Central Highlands | 1 (1.2%) |
|  |  | Southern Vietnam | South East | 41 (5.3%) |
|  |  |  | Mekong River Delta | 28 (5.7%) |
|  | **2b** | Southern Vietnam | Mekong River Delta | 2 (0.4%) |
|  | **2i** | Central Vietnam | Central Highlands | 1 (1.2%) |
|  |  | Southern Vietnam | South East | 1 (0.1%) |
|  |  |  | Mekong River Delta | 1 (0.2%) |
|  | **2j** | Southern Vietnam | Mekong River Delta | 4 (0.8%) |
|  | **2m** | Southern Vietnam | South East | 15 (1.9%) |
|  |  |  | Mekong River Delta | 44 (9%) |
|  | **2t** | Southern Vietnam | Mekong River Delta | 1 (0.2%) |
| **3** | **3a** | Northern Vietnam | Red River Delta | 4 (2.2%) |
|  |  | Central Vietnam | Central Highlands | 2 (2.4%) |
|  |  | Southern Vietnam | South East | 2 (0.3%) |
|  |  |  | Mekong River Delta | 2 (0.4%) |
|  | **3b** | Northern Vietnam | North Mountains and Midlands | 8 (8.7%) |
|  |  |  | Red River Delta | 7 (3.9%) |
|  |  | Central Vietnam | Central Highlands | 3 (3.6%) |
|  |  | Southern Vietnam | South East | 3 (0.4%) |
| **6** | **6a** | Northern Vietnam | North Mountains and Midlands | 32 (34.8%) |
|  |  |  | Red River Delta | 76 (42%) |
|  |  | Central Vietnam | North Central Coast | 3 (13.6%) |
|  |  |  | Central Highlands | 21 (25%) |
|  |  | Southern Vietnam | South East | 201 (25.8%) |
|  |  |  | Mekong River Delta | 135 (27.5%) |
|  | **6c** | Northern Vietnam | Red River Delta | 1 (0.6%) |
|  | **6e** | Northern Vietnam | North Mountains and Midlands | 7 (7.6%) |
|  |  |  | Red River Delta | 11 (6.1%) |
|  |  | Central Vietnam | North Central Coast | 4 (18.2%) |
|  |  |  | Central Highlands | 7 (8.3%) |
|  |  | Southern Vietnam | South East | 113 (14.5%) |
|  |  |  | Mekong River Delta | 101 (20.6%) |
|  | **6h** | Northern Vietnam | North Mountains and Midlands | 6 (6.5%) |
|  |  |  | Red River Delta | 9 (5%) |
|  |  | Central Vietnam | Central Highlands | 3 (3.6%) |
|  |  | Southern Vietnam | South East | 3 (0.4%) |
|  |  |  | Mekong River Delta | 17 (3.5%) |
|  | **6i** | Central Vietnam | North Central Coast | 1 (4.5%) |
|  | **6k** | Northern Vietnam | North Mountains and Midlands | 2 (2.2%) |
|  | **6l** | Northern Vietnam | North Mountains and Midlands | 2 (2.2%) |
|  |  | Central Vietnam | Central Highlands | 1 (1.2%) |
|  |  | Southern Vietnam | South East | 19 (2.4%) |
|  |  |  | Mekong River Delta | 18 (3.7%) |
|  | **6o** | Central Vietnam | Central Highlands | 1 (1.2%) |
|  |  | Southern Vietnam | South East | 5 (0.6%) |
|  |  |  | Mekong River Delta | 5 (1%) |
|  | **6p** | Southern Vietnam | South East | 2 (0.3%) |
|  |  |  | Mekong River Delta | 4 (0.8%) |
|  | **6q** | Northern Vietnam | Red River Delta | 1 (0.6%) |
|  | **6t** | Central Vietnam | Central Highlands | 2 (2.4%) |
|  |  | Southern Vietnam | South East | 1 (0.1%) |
|  | **6u** | Central Vietnam | Central Highlands | 3 (3.6%) |
|  |  | Southern Vietnam | South East | 5 (0.6%) |
|  |  |  | Mekong River Delta | 1 (0.2%) |
|  | **6xb** | Southern Vietnam | South East | 3 (0.4%) |
|  |  |  | Mekong River Delta | 3 (0.6%) |

***Supplementary Table 3.*** ***Baseline demographic and clinical characteristics of patients stratified by HCV genotype.*** *Data are presented as median [interquartile range, Q1–Q3] for continuous variables (age and viral load) and as frequency (n/N, %) for categorical variables. The denominator (N) for HIV infection, HBV infection, and cirrhosis status vary due to the availability of clinical data for these specific parameters. P-values indicate the statistical significance of differences across the four genotype groups. * P-values were calculated using the Kruskal–Wallis rank sum test. † P-values were calculated using Pearson’s Chi-squared test.*

| **Characteristic** | **Total** N = 1,649 | **Genotype 1** N = 649 | **Genotype 2** N = 140 | **Genotype 3** N = 31 | **Genotype 6** N = 829 | **p-value** |
| --- | --- | --- | --- | --- | --- | --- |
| **Age, Median [Q1–Q3]** | 48 [38–59] | 42 [36–53] | 56 [49–63] | 36 [30–46] | 51 [40–61] | **2.7e-31*** |
| **Sex, n/N (%)** |  |  |  |  |  | **6.8e-09†** |
| Female | 697/1,649 (42.3%) | 226/649 (34.8%) | 83/140 (59.3%) | 7/31 (22.6%) | 381/829 (46.0%) |  |
| Male | 952/1,649 (57.7%) | 423/649 (65.2%) | 57/140 (40.7%) | 24/31 (77.4%) | 448/829 (54.0%) |  |
| **HIV infection, n/N (%)** |  |  |  |  |  | **3.2e-07†** |
| Positive | 223/926 (24.1%) | 131/399 (32.8%) | 15/54 (27.8%) | 5/26 (19.2%) | 72/447 (16.1%) |  |
| Negative | 703/926 (75.9%) | 268/399 (67.2%) | 39/54 (72.2%) | 21/26 (80.8%) | 375/447 (83.9%) |  |
| **HBV infection, n/N (%)** |  |  |  |  |  | 0.51† |
| Positive | 135/1,484 (9.1%) | 53/573 (9.2%) | 16/131 (12.2%) | 3/29 (10.3%) | 63/751 (8.4%) |  |
| Negative | 1349/1,484 (90.9%) | 520/573 (90.8%) | 115/131 (87.8%) | 26/29 (89.7%) | 688/751 (91.6%) |  |
| **Cirrhosis, n/N (%)** |  |  |  |  |  | 0.06† |
| Cirrhosis | 180/1,223 (14.7%) | 55/432 (12.7%) | 19/113 (16.8%) | 0/23 (0.0%) | 106/655 (16.2%) |  |
| Absence of Cirrhosis | 1043/1,223 (85.3%) | 377/432 (87.3%) | 94/113 (83.2%) | 23/23 (100.0%) | 549/655 (83.8%) |  |
| **Log Baseline Viral Load, Median [Q1–Q3]** | 6.37 [5.62–6.90] | 6.42 [5.77–6.96] | 6.46 [5.75–6.82] | 6.35 [5.32–6.75] | 6.34 [5.56–6.90] | 0.36* |
| * Kruskal–Wallis rank sum test; † Pearson’s Chi-squared test | | | | | | |

***Supplementary Table 4. Prevalence of RASs to DAAs across HCV subtypes.*** *Each value represents the percentage of samples carrying the variant in the indicated HCV subtype within this Vietnamese cohort.* ***Abbreviations:*** *GLE - glecaprevir; GZR - grazoprevir; VOX - voxilaprevir; DCV - daclatasvir; EBV - elbasvir; LDV - ledipasvir; PIB - pibrentasvir; VEL - velpatasvir; SOF - sofosbuvir.*

| **RAS** | **Protein** | **Resistant to DAA** | **1a** | **1b** | **2a** | **2b** | **2m** | **3a** | **3b** | **6a** | **6e** |
| --- | --- | --- | --- | --- | --- | --- | --- | --- | --- | --- | --- |
| V36L | NS3 | GZR/VOX | 0.7 (2/307): GZR/VOX | 3.7 (10/273): GZR |  |  |  |  |  |  |  |
| V36M | NS3 | GLE/GZR | 0.3 (1/307) |  |  |  |  |  |  |  |  |
| Y56F | NS3 | GZR |  | 4.0 (11/273) |  |  |  |  |  |  |  |
| Q80K | NS3 | GZR/VOX | 54.8 (170/310) |  |  |  |  |  |  |  |  |
| Q80L | NS3 | GZR/VOX | 1.9 (6/310): VOX | 2.6 (7/269): GZR |  |  |  |  |  |  |  |
| Q80R | NS3 | GZR | 0.3 (1/310) | 0.4 (1/269) |  |  |  |  |  |  |  |
| R155W | NS3 | VOX | 0.3 (1/304) |  |  |  |  |  |  |  |  |
| A156G | NS3 | GLE/GZR | 0.3 (1/306) |  |  |  |  |  |  |  |  |
| A156L | NS3 | GZR/VOX | 0.3 (1/306) |  |  |  |  |  |  |  |  |
| A156S | NS3 | GZR | 0.3 (1/306) |  |  |  |  |  |  |  |  |
| A156V | NS3 | GLE/GZR/VOX | 0.3 (1/306) |  |  |  |  |  |  |  |  |
| D168A | NS3 | GLE/GZR/VOX | 0.3 (1/311) |  |  |  |  |  |  |  |  |
| D168E | NS3 | GZR | 0.3 (1/311) | 1.1 (3/275) |  |  |  |  |  | 0.7 (3/414) |  |
| D168T | NS3 | GZR/VOX | 0.3 (1/311) |  |  |  |  |  |  |  |  |
| D168V | NS3 | GLE/GZR/VOX | 0.3 (1/311) |  |  |  |  |  |  |  |  |
| D168Y | NS3 | GLE/GZR |  |  |  |  |  |  |  | 0.2 (1/414) |  |
| V36L+Q80K | NS3 | GZR/VOX | 0.3 (1/302) |  |  |  |  |  |  |  |  |
| L28F | NS5A | DCV |  | 0.4 (1/279) |  |  |  |  |  | 43.3 (181/418) |  |
| L28M | NS5A | DCV/EBV/LDV/VEL |  | 1.1 (3/279): DCV/EBV/LDV |  |  |  |  |  | 0.5 (2/418): VEL |  |
| L28S | NS5A | VEL |  |  |  |  |  |  |  | 0.2 (1/418) |  |
| L28V | NS5A | DCV/VEL |  | 0.4 (1/279): DCV |  |  |  |  |  | 2.4 (10/418): VEL |  |
| M28A | NS5A | DCV/EBV/LDV/PIB/VEL | 0.3 (1/303) |  |  |  |  |  |  |  |  |
| M28T | NS5A | DCV/EBV/LDV/PIB/VEL | 0.7 (2/303) |  |  |  |  |  |  |  |  |
| M28V | NS5A | DCV/EBV/LDV/VEL | 4.0 (12/303) |  |  |  |  |  |  |  |  |
| A30K | NS5A | VEL |  |  |  |  |  |  | 95.0 (19/20) |  |  |
| Q30H | NS5A | DCV/EBV/LDV/VEL | 1.7 (5/303) |  |  |  |  |  |  |  |  |
| Q30R | NS5A | DCV/EBV/LDV/PIB/VEL | 1.7 (5/303) |  |  |  |  |  |  |  |  |
| R30L | NS5A | DCV |  | 0.4 (1/279) |  |  |  |  |  |  |  |
| R30Q | NS5A | DCV/EBV |  | 2.9 (8/279) |  |  |  |  |  |  |  |
| L31I | NS5A | VEL |  |  | 1.5 (1/65) |  |  |  |  |  |  |
| L31M | NS5A | DCV/EBV/LDV/PIB/VEL | 0.7 (2/302): DCV/EBV/LDV/PIB/VEL | 0.4 (1/280): DCV/EBV/LDV/VEL | 90.8 (59/65): DCV/EBV/VEL | 100.0 (2/2): DCV/VEL | 98.1 (53/54): DCV |  | 95.0 (19/20): VEL | 1.4 (6/417): DCV/EBV/VEL | 0.5 (1/220): DCV |
| L31V | NS5A | DCV/EBV/LDV/PIB/VEL |  | 0.4 (1/280) |  |  |  |  |  |  |  |
| T93S | NS5A | VEL |  |  |  |  |  |  |  | 1.9 (8/428) |  |
| Y93C | NS5A | DCV/EBV/LDV/VEL | 2.6 (8/304) |  |  |  |  |  |  |  |  |
| Y93F | NS5A | VEL |  |  | 3.0 (2/66) |  |  |  |  |  |  |
| Y93H | NS5A | DCV/EBV/LDV/PIB/VEL | 2.0 (6/304): DCV/EBV/LDV/PIB/VEL | 9.0 (25/277): DCV/EBV/LDV/PIB/VEL | 1.5 (1/66): DCV/EBV/VEL |  |  | 10.0 (1/10): DCV/EBV/LDV/PIB/VEL |  |  |  |
| Y93N | NS5A | DCV/EBV/LDV/PIB/VEL | 0.7 (2/304): DCV/EBV/LDV/PIB/VEL |  | 42.4 (28/66): DCV/VEL |  |  |  |  |  |  |
| Y93S | NS5A | EBV/LDV/VEL | 0.3 (1/304) |  |  |  |  |  |  |  |  |
| Y93T | NS5A | LDV/VEL | 1.6 (5/304) |  |  |  |  |  |  |  |  |
| A30K+L31M | NS5A | VEL |  |  |  |  |  |  | 90.0 (18/20) |  |  |
| Q30H+Y93H | NS5A | DCV/EBV/LDV/PIB/VEL | 0.7 (2/294) |  |  |  |  |  |  |  |  |
| S282R | NS5B | SOF | 1.0 (3/310) |  |  |  |  |  |  |  |  |
| C289L | NS5B | SOF | 0.3 (1/313) | 0.4 (1/284) |  |  |  |  |  |  |  |
| C316H | NS5B | SOF | 0.3 (1/307) | 0.7 (2/281) |  |  |  |  |  |  |  |
| C316N | NS5B | SOF |  | 5.0 (14/281) |  |  |  |  |  |  |  |

***Supplementary Table 5. Prevalence of sub-clinical RASs to DAAs across HCV subtypes.*** *Each value represents the percentage of samples carrying the variant in the indicated HCV subtype within this Vietnamese cohort.* ***Abbreviations:*** *GLE - glecaprevir; GZR - grazoprevir; VOX - voxilaprevir; DCV - daclatasvir; EBV - elbasvir; LDV - ledipasvir; PIB - pibrentasvir; VEL - velpatasvir; SOF - sofosbuvir.*

| **RAS** | **Protein** | **Resistant to DAA** | **1a** | **1b** | **2a** | **2b** | **2i** | **2j** | **3a** | **6a** |
| --- | --- | --- | --- | --- | --- | --- | --- | --- | --- | --- |
| Q41K | NS3 | VOX |  |  |  |  |  |  |  | 0.5 (2/421) |
| Q41R | NS3 | VOX |  |  |  |  |  |  |  | 0.2 (1/421) |
| V55A | NS3 | GZR/VOX | 0.3 (1/309) |  |  |  |  |  |  |  |
| V107I | NS3 | GZR | 0.3 (1/311) | 0.4 (1/278) |  |  |  |  |  |  |
| S122G | NS3 | GZR | 2.0 (6/295) | 3.6 (10/276) |  |  |  |  |  |  |
| S122N | NS3 | GZR | 1.7 (5/295) |  |  |  |  |  |  |  |
| A166S | NS3 | GLE |  |  |  |  |  |  | 30.0 (3/10) |  |
| I170T | NS3 | GZR | 0.3 (1/312) |  |  |  |  |  |  |  |
| I170V | NS3 | GZR | 6.1 (19/312) |  |  |  |  |  |  |  |
| Q80K+I132V | NS3 | GZR | 1.4 (4/296) |  |  |  |  |  |  |  |
| S122T+V170I | NS3 | GZR |  | 0.4 (1/269) |  |  |  |  |  |  |
| Y56F+S122N | NS3 | VOX |  | 0.4 (1/264) |  |  |  |  |  |  |
| Y56F+S122N+V170I | NS3 | VOX |  | 0.4 (1/262) |  |  |  |  |  |  |
| Y56F+V170I | NS3 | GZR |  | 2.3 (6/265) |  |  |  |  |  |  |
| K24R | NS5A | DCV/LDV/PIB/VEL | 1.0 (3/302) |  |  |  |  |  |  |  |
| Q24H | NS5A | DCV |  |  |  |  |  |  |  | 0.2 (1/415) |
| Q24K | NS5A | DCV/EBV/LDV/PIB/VEL |  | 1.8 (5/280) |  |  |  |  |  |  |
| T24A | NS5A | DCV |  |  | 6.2 (4/65) |  |  |  |  |  |
| T24S | NS5A | DCV |  |  | 3.1 (2/65) |  |  |  |  |  |
| P29R | NS5A | PIB | 0.3 (1/303) |  |  |  |  |  |  |  |
| P32L | NS5A | DCV/LDV/VEL |  |  |  |  |  |  |  | 0.2 (1/417) |
| P32S | NS5A | DCV/LDV |  | 1.4 (4/281) |  |  |  |  |  |  |
| F37I | NS5A | DCV |  | 1.4 (4/282) |  |  |  |  |  |  |
| F37L | NS5A | DCV |  | 18.1 (51/282) |  |  |  |  |  |  |
| Y54L | NS5A | DCV |  | 2.8 (8/286) |  |  |  |  |  |  |
| H58D | NS5A | DCV/EBV/LDV/PIB/VEL | 1.0 (3/311) |  |  |  |  |  |  |  |
| H58P | NS5A | DCV | 5.1 (16/311) |  |  |  |  |  |  |  |
| H58R | NS5A | DCV | 1.6 (5/311) |  |  |  |  |  |  |  |
| P58A | NS5A | DCV |  | 0.4 (1/284) |  |  |  |  |  |  |
| P58L | NS5A | DCV |  | 0.7 (2/284) |  |  |  |  |  |  |
| P58S | NS5A | DCV/VEL |  | 3.9 (11/284): DCV |  | 50.0 (1/2): VEL |  |  |  |  |
| T58A | NS5A | DCV |  |  |  |  |  |  |  | 1.4 (6/425) |
| T58G | NS5A | VEL |  |  |  |  |  |  |  | 0.2 (1/425) |
| T58H | NS5A | VEL |  |  |  |  |  |  |  | 0.2 (1/425) |
| T58S | NS5A | DCV |  |  |  |  |  |  |  | 1.9 (8/425) |
| A62L | NS5A | DCV |  |  |  |  |  |  | 20.0 (2/10) |  |
| E62A | NS5A | PIB | 0.3 (1/311) |  |  |  |  |  |  |  |
| E62D | NS5A | DCV |  | 4.2 (12/283) |  |  |  |  |  |  |
| A92T | NS5A | LDV/VEL | 0.3 (1/305): LDV | 2.2 (6/277): LDV |  |  |  |  |  | 0.2 (1/429): VEL |
| C92S | NS5A | DCV/VEL |  |  | 4.5 (3/67) |  |  |  |  |  |
| F37L+Y54H | NS5A | DCV |  | 5.4 (15/280) |  |  |  |  |  |  |
| F37L+Y93H | NS5A | DCV |  | 1.8 (5/273) |  |  |  |  |  |  |
| L31M+Y54H | NS5A | LDV |  | 0.4 (1/278) |  |  |  |  |  |  |
| Q24K+L28M | NS5A | DCV |  | 1.1 (3/278) |  |  |  |  |  |  |
| Q24K+L28M+R30Q | NS5A | DCV |  | 1.1 (3/278) |  |  |  |  |  |  |
| T24S+L31M | NS5A | DCV/VEL |  |  | 3.1 (2/65): VEL |  | 66.7 (2/3): DCV | 75.0 (3/4): VEL |  |  |
| Y54H+Y93H | NS5A | DCV |  | 1.8 (5/275) |  |  |  |  |  |  |
| A150V | NS5B | SOF |  |  |  |  |  |  | 40.0 (4/10) |  |
| K206E | NS5B | SOF |  |  |  |  |  |  | 10.0 (1/10) |  |
| E237G | NS5B | SOF | 10.7 (34/317) |  |  |  |  |  |  |  |

***Supplementary Table 6. Summary of clinical studies included in the HCV cohort.*** *Overview of the six clinical studies contributing HCV positive participants to the cohort between 2013 and 2023 in Viet Nam. The table summarises study periods, inclusion and exclusion criteria, enrolment sites, and the number of HCV infected participants enrolled in each study. Participants were recruited through hospital-based clinical studies and trials conducted across multiple regions of Viet Nam.*

| **Study** | **Study period** | **Inclusion criteria** | **Exclusion criteria** | **Enrolment sites** | **Number of HCV positive participants enrolled** |
| --- | --- | --- | --- | --- | --- |
| **VHARP001** | 2013 - 2015 | • HIV infected adults ≥18 • Meet Vietnam guideline to begin ART (CD4 count < 350 cells/mm3 and/or WHO stage III or IV disease) • Hepatitis C infection • AST and ALT ≤ 2 x ULN (≤ 80 U/L) • Estimated creatinine clearance ≥ 60 mL/min | • Prior ART • Positive Hepatitis B surface antigen • Clinical evidence of de-compensated cirrhosis • Requirement for acute therapy for other AIDS-defining illness within 14 days prior to study entry • Currently on rifampicin therapy • In the first trimester of pregnancy, intent to become pregnant, or breast feeding during the study period | • Southern Viet Nam: Ho Chi Minh city | 79 participants |
| **VIZIONS** | 2014 - 2016 | • Hospital surveillance recruits patients based on predefined clinical syndromes: CNS/enteric/jaundice/respiratory syndromes • Frequent occupational or residential animal exposure, belonging to predefined high-risk groups: farmers/farm households, slaughterers, animal health workers, other animal-exposed occupations • Willing to participate in longitudinal follow-up and sampling • Undiagnosed jaundice patients • Clinical diagnosis of acute respiratory infection syndrome • Fever or recent history of fever | • Previously enrolled in the study • Recent hospitalization • Symptoms not due to infectious cause • Not resident in the hospital catchment province | • Southern Viet Nam: Ho Chi Minh city, Dong Thap  • Central Viet Nam: Dak Lak, Khanh Hoa, Thua Thien Hue | 87 participants |
| **01EI** | 2014 - 2020 | • Age ≥1 year • Attending at hepatology outpatient or inpatient department of Hospital for Tropical Diseases • Have visual signs of jaundice, or a total bilirubin of >50 mmol/l or 3 mg/dl and/or patient have a high serum alanine aminotransferase (ALT), defined as > 80 U/L. | • Previously enrolled in the study • Hepatitis of known etiology. • Liver cirrhosis/hepatocellular carcinoma/terminal illness • Drug-induced hepatitis • Have extra-hepatic cholestasis (obstructive jaundice) or known to have an alternative non-infectious diagnosis | • Southern Viet Nam: Ho Chi Minh city | 317 participants |
| **KAPB** | 2018 - 2020 | • Age ≥18 • Attending the out-patient department at the Hospital for Tropical Diseases for HCV care • Previously diagnosed with HCV Infection | • Previous treatment with DAA drugs • Enrolled in any other intervention trial | • Southern Viet Nam: Ho Chi Minh city | 333 participants |
| **SEARCH-1** | 2019 - 2020 | • Age ≥18 • At least one detectable viraemia prior to the screening visit, with no intervening undetectable results • Plasma HCV RNA >LLOQ at screening • BMI >=18kg/m^2^  • Laboratory tests: creatinine clearance (estimated using Cockcroft-Gault) >=60ml/min, international normalised ratio (INR) <1.5xULN | • Prior HCV treatment • HIV/HBV infection • Pregnancy • Severe disease • Solid organ malignancy within 5 years prior to screening • Disorder which may cause ongoing liver disease including, but not limited to, ongoing alcohol misuse • Use of contraindicated medications • Use of other investigational products in clinical studies within 60 days of screening • History of severe pre-existing cardiac disease, including unstable or uncontrolled cardiac disease, in the previous six months • Patients currently using amiodarone or other anti-arrhythmics (including patients with permanent pacemakers). • Pregnancy  • Symptomatic Haemoglobinopathy (e.g., thalassemia major, sickle-cell anaemia). • Unlikely to be able to attend follow-up visits for any reason | • Southern Viet Nam: Ho Chi Minh city | 93 participants |
| **VIETNARMS** | 2020 - 2023 | • Age ≥18 • Prior evidence of Hepatitis C virus infection for more than 6 months AND at least one detectable (>LLOQ) HCV viraemia within 60 days prior to the enrolment visit, with no subsequent undetectable results • Laboratory tests at the screening visit or within 60 days of enrolment/randomisation:  a.Creatinine clearance (estimated using Cockcroft-Gault) ≥50ml/min b.TSH within reference range (0.4-4.0mIU/L) c.Haemoglobin >8.5 g/dL • Mild liver disease | • Prior DAA failure • Unidentified HCV genotype after repeated sequencing attempts • Severe disease • Disorder which may cause ongoing liver disease including, but not limited to, ongoing alcohol misuse • Use of contraindicated medications • Currently using amiodarone or digoxin • Use of other investigational products in clinical studies within 60 days of screening | • Southern Viet Nam: Ho Chi Minh city  • Northern Viet Nam: Hanoi | 95 participants screened but not enrolled 645 participants enrolled |

***Supplementary Table 7. Primers used for amplification of HCV genomic regions by Sanger sequencing.*** *Primer names indicate the target genomic region, the fragment number when a region was amplified in multiple overlapping fragments, the primer direction (F, forward; R, reverse), and the PCR round when nested PCR was used (1, outer PCR; 2, nested PCR). Alternative primers designed for the same amplification step are indicated by the suffix “a”. Primer sequences are shown in the 5′–3′ orientation. Degenerate bases follow the IUPAC nucleotide code.*

| **Gene** | **Primer name** | **Sequence (5′–3′)** |
| --- | --- | --- |
| 5'UTR-Core | Core-F1 | GGGGCGACACTCCRCCATG |
| 5'UTR-Core | Core-R1 | GCANGASAGCAGDGCCAGNAGG |
| 5'UTR-Core | Core-F2 | CACTCCCCTGTGAGGAACTWCTG |
| 5'UTR-Core | Core-R2 | GAAGATAGARAAARAGCAAC |
| E1 | E1-F1 | GCAACAGGGAAYYTDCCTGG |
| E1 | E1-R1 | GGNGTGAARCAGTABACAGGNCC |
| E1 | E1-F2 | CCYGGTTGCTCYTTYTCTATC |
| E1 | E1-R2 | GGNGCATARTGCCAGCARTABGG |
| E2-p7 | p7-F1 | GCGGNCCDGTVTACTGYTTCAC |
| E2-p7 | p7-R1 | CRTCVCKVCCNCCBCKDRC |
| E2-p7 | p7-R1a | CCACWCNTGCANBDBGGCCTC |
| E2-p7 | p7-F2 | CCTGTDGTSGTVGGNACBACYG |
| E2-p7 | p7-R2 | GVHBANGADRTABBGNADCCNC |
| NS2 | NS2-F1 | CAVGYNGANGCVGCVKTGGAG |
| NS2 | NS2-R1 | CGGCVCCRTGRWRNRYNSYCC |
| NS2 | NS2-F2 | GAGARBCTBRTARTBCTBAAYG |
| NS2 | NS2-R2 | GCNARNVNGGWNYKDGTRRC |
| NS3 | NS3F1-F1 | GGBTGGMRGCTBCTNGCBCC |
| NS3 | NS3F1-R1 | CCVGGRGGNGTGGCBGTBGC |
| NS3 | NS3F1-R1a | GGNGTNGCBGTBGCGARVAC |
| NS3 | NS3F1-F2 | CSATCACNGCNTAYGCYCARC |
| NS3 | NS3F1-R2 | GCYTGGTCNARVACNGTNCC |
| NS3 | NS3F2-F1 | GGCAARTTYCTBGCNGATGG |
| NS3 | NS3F2-R1 | GMRCAYTCYTCCATCTCRTC |
| NS3 | NS3F2-F2 | GRCAYCTBATTTTCTGYCATTC |
| NS3 | NS3F2-F2a | GAAGAARTGYGAYGAGCTSGC |
| NS3 | NS3F2-R2 | GCARTAVGCKGCBARAGCNGC |
| NS4A-NS4B | NS4-F1 | CNAGYACVTGGGTBHTVGTBG |
| NS4A-NS4B | NS4-R1 | GTGNGTNGGKGANACRTGGTTNCC |
| NS4A-NS4B | NS4-F2 | GAKGAGATGGARGAGTGCKC |
| NS4A-NS4B | NS4-R2 | CGAABGCTATNAGYCKRTTCATCCAC |
| NS5A | NS5AF1-F1 | GAYRTNTGGGABTGGATATG |
| NS5A | NS5AF1-R1 | GGHGCNGATABCTGGCTNGC |
| NS5A | NS5AF1-F2 | GGCTBNVNGYYAARCTYNNNHN |
| NS5A | NS5AF1-R2 | GABAGCTGGCTNGCBGADGAGC |
| NS5A | NS5AF2-F1 | GGCARGAGATGGGHRGCAAYATCAC |
| NS5A | NS5AF2-R1 | GAGTCAAARCANCKGGTRTC |
| NS5A | NS5AF2-F2 | GVRTNGAGTCTGAVAMCAARG |
| NS5A | NS5AF2-R2 | CNGGRTANACDATNWVVCGDGC |
| NS5A | NS5AF2-R2a | CYTCRTTCTTNGCCATRATDG |
| NS5B | NS5B-F1 | CCASATCARCTCCGTSTGGCAG |
| NS5B-3'UTR | NS5B-T-R | AAAAAAAAAAAAAAAAAARR |
| NS5B | NS5B-R1a | GGVGCRWANWKGATGATNBWBCC |
| NS5B | NS5B-F2 | GTGTGGGAGGACYTBCTGGAAGAC |
| NS5B | NS5B-R1 | GTGDCKDGCTGYCTCCCAVGC |
| NS5B | NS5B-R2 | GTCATRGCHTCCGTGAADGCTC |
